# Anchoring Redox Mediator on COFs for Efficient Solar to Hydrogen Conversion

**DOI:** 10.1002/adma.202510193

**Published:** 2025-08-27

**Authors:** Haijun Hu, Xiaodong Sun, Yali Ma, Hui Li, Wei Zhang, Hua Fan, Hongwei Huang, Tianyi Ma

**Affiliations:** ^1^ Institute of Clean Energy Chemistry, Key Laboratory for Green Synthesis and Preparative Chemistry of Advanced Materials, College of Chemistry Liaoning University Shenyang 110036 P. R. China; ^2^ Centre for Atomaterials and Nanomanufacturing (CAN), School of Science RMIT University Melbourne VIC 3000 Australia; ^3^ College of Chemical Engineering Shenyang University of Chemical Technology Shenyang 110142 P. R. China; ^4^ ARC Industrial Transformation Research Hub for Intelligent Energy Efficiency in Future Protected Cropping (E2Crop) Melbourne VIC 3000 Australia; ^5^ Aqualux AU Pty Ltd 12 Kanangra Cres Clontarf NSW 2093 Australia; ^6^ Beijing Key Laboratory of Materials Utilization of Nonmetallic Minerals and Solid Wastes, National Laboratory of Mineral Materials, School of Materials Science and Technology China University of Geosciences Beijing 100083 P. R. China

**Keywords:** covalent organic frameworks, photocatalysis, redox mediator

## Abstract

To address severe carrier recombination in Z‐scheme heterojunctions, redox mediators such as IO_3_^−^/^I−^ or Fe^3^⁺/Fe^2^⁺ are often introduced, yet their dispersion in solution causes instability, low electron transport efficiency and side reactions. Herein, an innovative Fe‐coordinated 2D Z‐scheme heterojunction composed of TpPa‐1‐COF (TP1C) and Bi_2_WO_6_ (BWO) is developed for efficient photocatalytic H_2_ production. Unlike traditional indirect Z‐scheme heterojunctions, the Fe^3+^/Fe^2+^ mediator is firmly anchored on the skeleton of COFs, thus enhancing recyclability, charge migration and long‐lasting stability, which is supported by extended X‐ray absorption fine structure (EXAFS) and a range of electrochemical tests. In addition, the formation of 2D Z‐scheme heterojunctions not only retains high redox properties but also provides abundant active sites for photocatalytic reactions. Consequently, the photocatalytic H_2_ production rate of 25% BWO/Fe/TP1C reaches up to 6.31 mmol·g^−1^·h^−1^ without the addition of co‐catalysts, being about 28.68 times as high as that of pure COFs and 2.3 folds over that of 25% BWO/TP1C, exceeding a host of COF‐based photocatalysts. The findings of this research highlight the potential of novel indirect Z‐scheme heterojunctions for advanced photocatalytic applications, offering a new pathway to overcome the limitations of traditional COF‐based systems in hydrogen production.

## Graphical abstract

In this work, Fe ions are first fixed on COFs skeleton to construct a novel 2D Z‐scheme heterojunction (BWO/Fe/TP1C). The cycling of Fe^3+^/Fe^2+^ significantly improves the electron transfer rate. In addition, the construction of 2D Z‐scheme heterojunction not only promotes the separation of photogenerated carriers but also ensures its high redox activity. As a result, 25% BWO/Fe/TP1C demonstrates exceptional photocatalytic performance, achieving a hydrogen evolution rate of 6.31 mmol·g^−1^·h^−1^, which surpasses most COF‐based photocatalysts.

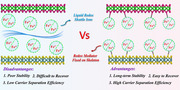

## 1 Introduction

The challenges of resource depletion and environmental pollution are becoming increasingly severe. Hydrogen energy has emerged as a promising solution to these pressing issues.^[^ ^1^, ^2^, ^3^, ^4^, ^5^ ^]^ Hence, the pursuit of stable and high‐activity photocatalysts for H_2_ production is of great significance. Covalent Organic Frameworks (COFs) are a class of crystalline, porous materials constructed from organic building blocks linked through covalent bonds.^[^ ^6^, ^7^, ^8^, ^9^, ^10^ ^]^ They possess tunable structures, large surface areas, and excellent thermal and chemical stability, making them ideal candidates for various applications, especially in the field of photocatalysis.^[^ ^11^, ^12^, ^13^, ^14^, ^15^ ^]^ In photocatalysis, COFs offer several advantages, such as efficient light absorption, adjustable band gaps, and abundant active sites for catalytic reactions.^[^ ^16^, ^17^, ^18^ ^]^ Additionally, their ordered porosity facilitates the transport of reactants and products, enhancing overall catalytic efficiency. However, the application of COFs in photocatalysis is significantly hindered by the severe recombination of photogenerated charge carriers. This rapid recombination reduces the number of electrons and holes available for redox reactions, limiting the photocatalytic efficiency.^[^ ^19^, ^20^, ^21^, ^22^, ^23^ ^]^ The construction of heterojunctions is a commonly used and effective method to improve the photocatalytic performance of COFs, which has attracted widespread attention in the scientific community, especially in the field of Z‐scheme heterojunctions.^[^ ^24^, ^25^, ^26^, ^27^ ^]^

He and coworkers used a one pot thermal method to integrate MOFs with COFs materials for photocatalytic applications. The prepared NH_2_‐MIL‐125(Ti)/TTB‐TTA hybrid material exhibited enhanced photocatalytic activity because the formation of direct Z‐scheme heterojunctions further promoted separation of charge carriers while ensuring the redox properties of the catalyst.^[^ ^24^ ^]^ However, for direct Z‐scheme heterojunctions, the process of photoexcited carrier movement across the interface of materials is often hindered by surface relaxation and the recombination of charge carriers inside each component. To overcome these challenges, the electron transfer mediator was introduced into Z‐scheme heterojunction. Liu and coworkers reported a reduced graphene oxide (rGO)‐mediated C_3_N_4_/C_3_N_5_ Z‐scheme heterojunction, in which rGO served as electron mediator.^[^ ^28^ ^]^ This configuration achieved highly efficient photogenerated charge separation, consequently demonstrating exceptional photocatalytic hydrogen production activity. However, this type of all‐solid‐state Z‐scheme heterojunction is susceptible to photo‐corrosion or chemical corrosion issues. Moreover, insufficient physical contact between the solid‐state electron mediator and semiconductor materials, combined with potential inadequate conductivity of the mediator, may hinder Z‐scheme charge transfer. In addition, some redox shuttle ions were also used to create Z‐scheme heterojunctions.^[^ ^29^ ^]^ Domen and his team built up a Z‐scheme system with IO_3_^−^/I^−^ as redox mediator for photocatalytic water splitting.^[^ ^30^ ^]^ As a consequence, the unfavorable backward reactions were substantially reduced, and the synthesized catalysts exhibited excellent photocatalytic activity with apparent quantum efficiency (AQE) of 6.3% at 420 nm. Moreover, Sasaki and his coworkers biult up a Z‐scheme photocatalytic system consist of Ru/SrTiO_3_:Rh and BiVO_4_ with [Co(bpy)_3_]^3+/2+^ and [Co(phen)_3_]^3+/2+^ as electron mediators.^[^ ^31^ ^]^ With the help of redox shuttle ions, the rate of charge transfer was significantly increased, thus the synthesized materials exhibited commendable water splitting activity in neutral pH conditions. In the context of liquid‐phase indirect Z‐scheme heterojunctions, while the incorporation of redox‐active shuttle ions enhanced the rate of electron transfer at the interfaces of the materials, it posed challenges in sustaining their long‐term stability and maintaining their active properties. In addition, the construction process of heterojunctions usually faces the problem of dimensional mismatch, which is unfavorable for electron migration and a large number of active sites. Besides, Dong et al. anchored single‐atom Pt onto the framework of COFs to enhance their photocatalytic activity.^[^ ^32^ ^]^ However, due to the scarcity of Pt resources, even single‐atom dispersion cannot fully address cost concerns, necessitating the exploration of non‐precious metal alternatives.

**Scheme 1:**
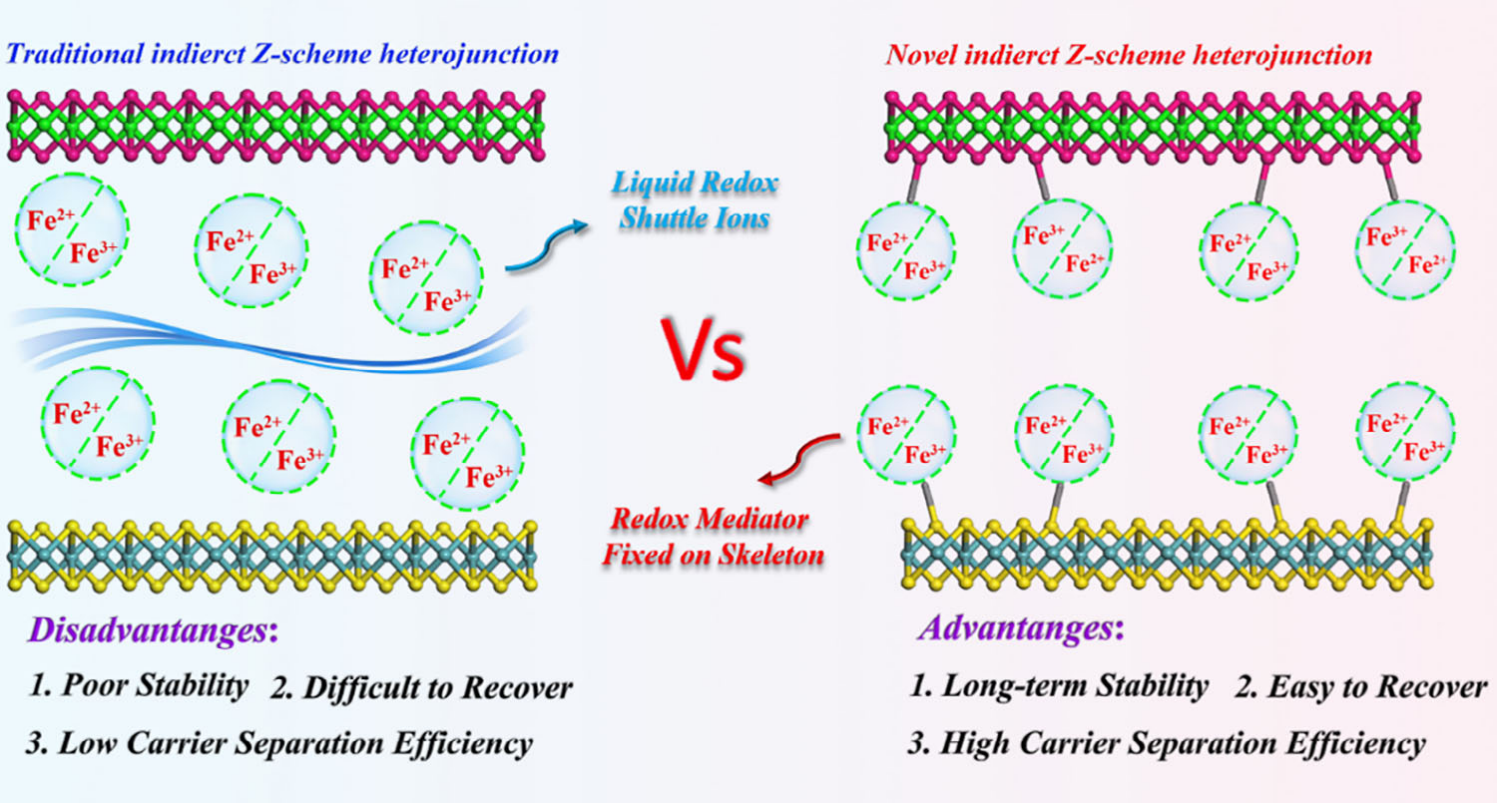
Comparison between traditional indirect Z‐scheme heterojunction and novel indirect Z‐scheme heterojunction.

To achieve excellent photocatalytic performance based on COFs materials, we first immobilized Fe ions into a novel 2D Z‐scheme heterojunction Bi_2_WO_6_/Fe/TpPa‐1‐COF (BWO/Fe/TP1C) for high‐efficient photocatalytic H_2_ production. The Fe ions were fixed on the skeleton of COFs as electron mediators, which can maintain long‐term stability and activation state compared to shuttle ions in the liquid phase. The Fe ions undergo cyclic valence state changes (Fe^3+^/Fe^2+^) during the photocatalytic process, further promoting interfacial charge transfer between materials and enabling it to achieve high photocatalytic activity. Furthermore, due to the unique charge transfer pathway, Z‐scheme heterojunctions not only have high carrier separation efficiency, but also maintain high redox properties. Besides, the 2D system formed between two materials brings abundant active sites, strong interfacial interactions, shortened charge transfer paths, and low charge transfer resistance. Accordingly, the optimal 25% BWO/Fe/TP1C achieves a high photocatalytic H_2_ production rate (6.31 mmol·g^−1^·h^−1^), ≈28.68 times that of the initial COFs material, and ≈2.3 times higher than that of 25% BWO/TP1C, also surpassing a host of reported photocatalysts based on COFs materials. Therefore, compared with traditional indirect Z‐scheme heterojunctions relying on liquid‐phase redox shuttles, the novel developed Z‐scheme system with redox mediators anchored on its skeleton offers distinct advantages, including enhanced long‐term stability, facile catalyst recovery, and improved charge carrier separation efficiency (**Scheme** **1**).

## 2 Results and Discussion

**Figure 1:**
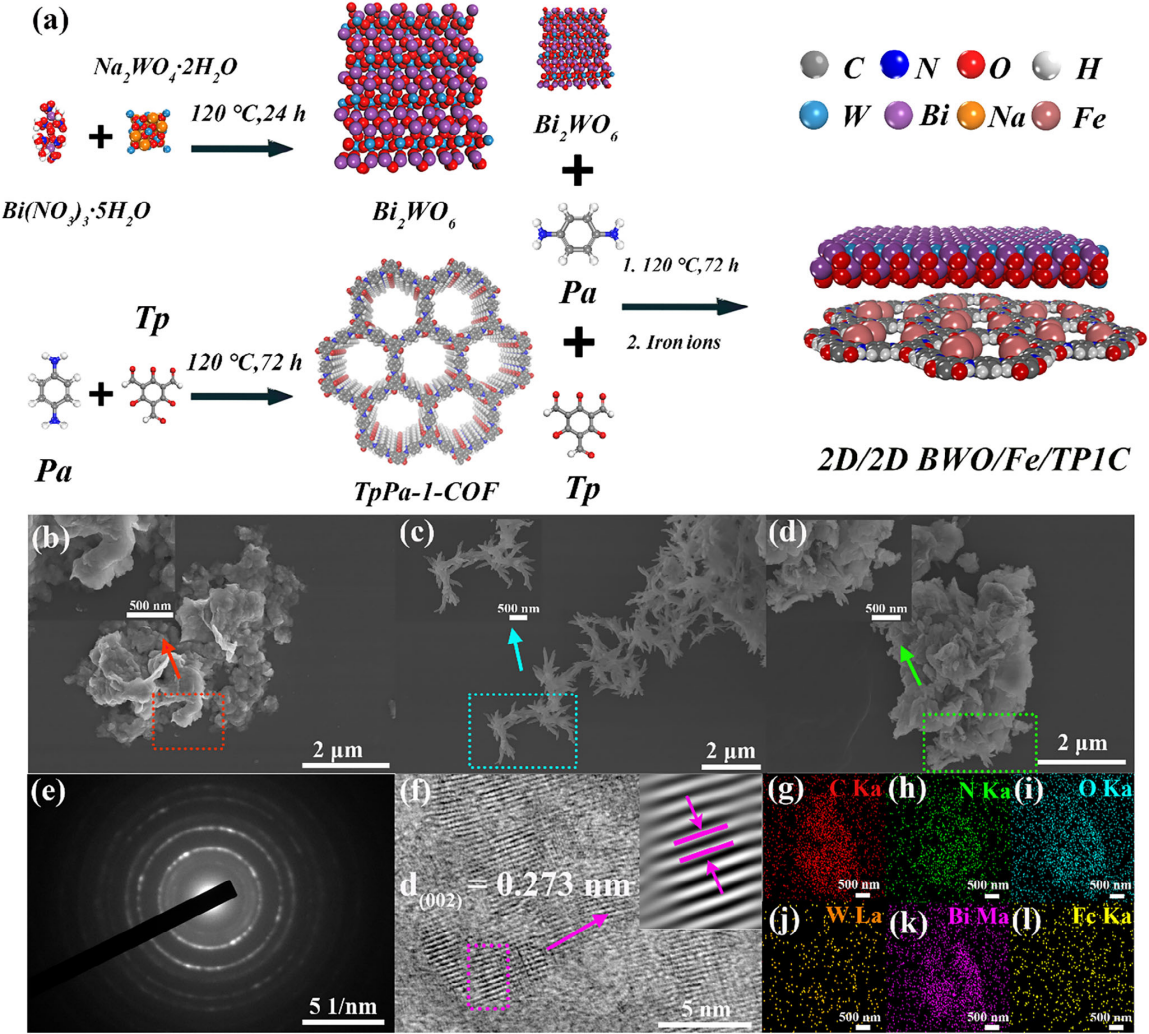
a) The preparation process for synthesized materials. b–d) The SEM measurements for BWO, TP1C and 25% BWO/Fe/TP1C. The inserts are corresponding SEM images at different magnifications. e) The SAED pattern of BWO. f) The HRTEM tests of BWO. g–l) Element distribution images of BWO/Fe/TP1C.

**Figure** **1**a visually depicted the preparation process of BWO/Fe/TP1C. First of all, the BWO nanosheets were synthesized by hydrothermal method using Na_2_WO_4_·2H_2_O and Bi(NO_3_)_3_·5H_2_O as the original materials. 1,3,5‐triformylphloroglucinol (Tp) and Paraphenylenediamine (Pa) were ground and then synthesized into TP1C through a solvothermal process. BWO/TP1C, synthesized by solvothermal method, was stirred in a solution of iron ions to obtain BWO/Fe/TP1C. The scanning electron microscopy (SEM) and transmission electron microscope (TEM) tests showed that BWO displayed nanosheet structure (Figure 1b; Figure S2, Supporting Information), and TP1C presented flower‐like morphology (Figure 1c; Figure S3, Supporting Information). In BWO/Fe/TP1C composite, the two materials were tightly bonded together (Figure 1d; Figure S4, Supporting Information). The selected area electron diffraction (SAED) pattern confirmed the polycrystalline structure of BWO (Figure 1e). Additionally, the lattice spacing measured in the high‐resolution transmission electron microscopy (HRTEM) image was ≈0.273 nm, which corresponded to the (002) crystal plane of BWO (Figure 1f). Moreover, as illustrated in Figure S5 (Supporting Information), the energy dispersive spectrometer (EDS) mapping images proved the presence of C, N, O, Fe, Bi, and W elements, further demonstrating the successful preparation of BWO/Fe/TP1C composite (Figure 1g–l).

Powder X‐ray Diffraction (PXRD) analysis (Figures S8 and S9, Supporting Information) confirmed the high purity and crystallinity of the synthesized BWO and TP1C, as their diffraction patterns matched well with the simulated peaks without detectable impurity phases.^[^ ^33^ ^]^ Besides, the Pawley refinement further validated the AA‐stacking model for TP1C, with refined parameters (P6/M, a = b = 22.56 Å, c = 3.40 Å, α = β = 90°, γ = 120°) demonstrating excellent agreement with experimental PXRD profiles (Figure S9, Supporting Information). For the BWO/Fe/TP1C composite, a distinct peak at 4.7° (attributed to the (100) plane of TP1C) was observed, while the remaining diffraction features corresponded to BWO, which demonstrated the successful integration of both materials while preserving their respective crystal structures during synthesis (Figure S10, Supporting Information). In addition, the thermogravimetric (TG) test results displayed that the weight of COFs and its hybrid materials decreased as temperature increased. Moreover, BWO/Fe/TP1C and TP1C began to decompose at around 420 and 450 °C, demonstrating their good thermal stability (Figure S11, Supporting Information).

**Figure 2:**
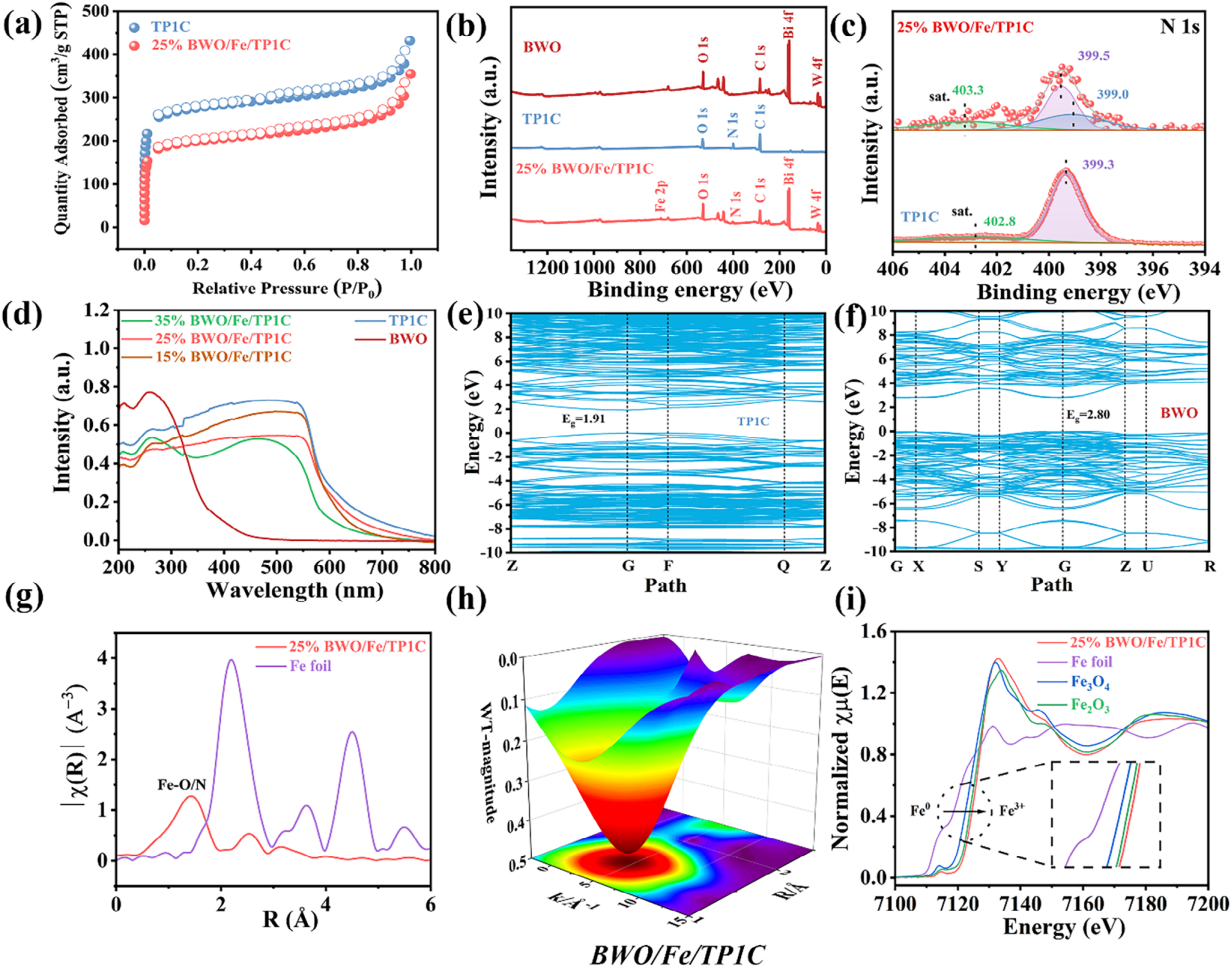
a) N_2_ adsorption/desorption isotherm curves of prepared samples. b) The XPS survey spectrum of prepared samples. c) The high‐revolution N 1s spectra. d) UV–vis diffuse reflectance spectra for prepared samples. e,f) Bandgap values for TP1C and BWO by DFT method. g) The r‐space distributions from the k^2^‐weighted Fe K‐edge EXAFS spectra of Fe foil and 25% BWO/Fe/TP1C (without phase correction). h) The wavelet analysis for 25% BWO/Fe/TP1C. i) Fe K‐edge XANES spectra of samples.

Based on the nitrogen adsorption/desorption tests at 77 K, TP1C and 25% BWO/Fe/TP1C composite both displayed type I adsorption behavior, characterized by a steep uptake at low relative pressures, indicative of microporous structures (**Figure** **2**a). As illustrated in Table S3 (Supporting Information), the Brunauer–Emmett–Teller (BET) surface areas of TP1C and 25% BWO/Fe/TP1C were determined to be 1035.8 m^2^/g and 750.9 m^2^/g, respectively. The reduced specific surface area was due to the coordination of Fe ions and the integration of BWO occupying the pores of the COFs materials. Obviously, the prepared materials possessed relatively high specific surface areas, creating rich reactive sites for photocatalytic reaction. Besides, Figures S12 and S13 (Supporting Information) showed the pore size distribution information of the prepared material, where the pore width of TP1C was ≈1.2 nm. According to the nitrogen adsorption experiment results, the pore volumes of 25% BWO/Fe/TP1C and TP1C can be obtained, which were 0.34 and 0.36 cm^3^·g^−1^, respectively.

X‐ray photoelectron spectroscopy (XPS) tests were carried out to analyze and characterize the chemical state and elemental composition of the synthesized products. Evidently, C, N, O, Fe, Bi, and W can be observed in XPS survey spectrum (Figure 2b). Then, the high‐resolution XPS tests illustrated the valence bonding situation. High‐resolution N 1s XPS spectra of pristine TP1C exhibited two distinct peaks at binding energies of 399.3 and 402.8 eV, where the higher‐energy peak corresponds to satellite peak (Figure 2c).^[^ ^34^ ^]^ Besides, for BWO/Fe/TP1C composite, the peak at 399.5 eV can be ascribed to the C─N bond resulting from the *β*‐ketoenamine linkage. The additional peak at 399.0 eV was attributed to the newly formed C═N bond, indicating the occurrence of the keto‐to‐enol transformation and the successful coordination of Fe ions with nitrogen atoms. Compared with the original COFs material, the binding energy peak position of BWO/Fe/TP1C showed an upshifted trend.

Figure S14 (Supporting Information) exhibited the high‐resolution C 1s spectrum of prepared materials, which can be divided into the three peaks at 284.6, 286.0, and 287.5 eV for BWO/Fe/TP1C, corresponding to the C═C, C─N, and C═O groups, respectively. The W 4f XPS spectra of BWO displayed two peaks at 35.0 and 37.2 eV, which stood for W 4f_7/2_ and W 4f_5/2_, proving the presence of W^6+^ in BWO (Figure S15, Supporting Information).^[^ ^35^ ^]^

Moreover, Figure S16 (Supporting Information) showed Bi 4f spectra of BWO, displaying two peaks at 158.8 and 164.1 eV, respectively. These binding energy peaks were referred to Bi 4f_7/2_ and Bi 4f_5/2_, indicating the presence of Bi^3+^.^[^ ^36^ ^]^ The binding energy peak position of BWO/Fe/TP1C was downshifted compared to that of bare BWO. The results of the XPS tests showed that Fe ions were successfully modified onto the skeleton of COFs and the binding energy peak shifts revealed the charge transfer path within the material, where electrons were transferred from TP1C to BWO in the absence of light. UV–vis diffuse reflectance spectroscopy (DRS) results showed that BWO exhibited a relatively poor light absorption competence (Figure 2d). In contrast, TP1C demonstrated a wider light absorption spectrum extending into the visible range. As a result, when BWO and COFs materials were combined, the composite material showed a pronounced red shift in its light absorption edge, leading to an improved light response capability. Tauc plots generated from Kubelka‐Munk function transformations provided reliable determination of the semiconductor bandgap values for both BWO and TP1C samples:

(1) $${\left(\alpha h\nu \right)}^{1/n}=A\left(h\nu -E_{g}\right)$$

in which E_g,_ ν, h, A, and α correspond to band gap energy, frequency, Planck's constant, proportional constant and absorption coefficient, respectively.^[^ ^37^ ^]^ For direct bandgap semiconductors, n = 1/2, whereas for indirect bandgap semiconductors, n = 2. Thus, optical bandgap analysis via Tauc plots were obtained (2.77 eV for BWO and 2.10 eV for TP1C) (Figures S17 and S18, Supporting Information). Besides, the theoretical bandgap values for TP1C and BWO were ≈1.91 and 2.80 eV, which were close to the experimental results (Figure 2e,f). The Mott–Schottky (M–S) plots in Figures S19 and S20 (Supporting Information) revealed positive slopes for both BWO and TP1C, confirming their n‐type semiconductor behavior. Based on the M‐S analysis, the flat‐band potentials (E_(Ag/AgCl)_) were estimated at ≈0.31 V for BWO and −0.69 V for TP1C. To further examine the normal hydrogen electrode (NHE) potential of the synthesized materials, the equation E_(NHE)_ = E_(Ag/AgCl)_ + E_0_ was applied, where E_0_ was ≈0.2 V. Based on this equation, the converted potentials relative to NHE were 0.51 V (BWO) and −0.49 V (TP1C). Consistent with established semiconductor principles, the lowest unoccupied molecular orbital (LUMO) or conduction band (CB) of n‐type materials typically resides ≈0.2 V above their E_(NHE)_ potential.^[^ ^38^ ^]^ Accordingly, the CB position of BWO was calculated at 0.31 V, while TP1C exhibited a LUMO level of −0.69 V (Figure S21, Supporting Information). Notably, the LUMO potential of TP1C were more negative than the H^+^/H_2_ redox potential and even exceeded the reduction potential of O_2_/·O_2_^−^ (−0.33 V vs NHE). Therefore, in the photocatalytic process, TP1C can facilitate the reduction of H^+^ in water to generate H_2_. Combining the CB/LUMO positions with the measured bandgap values, the valence band (VB) and highest occupied molecular orbital (HOMO) levels were derived as 3.08 V for BWO and 1.41 V for TP1C (Figure S21, Supporting Information). The appropriate bandgap alignment between BWO and TP1C made it feasible to form the Z‐scheme heterojunction. Moreover, the coordination environments of BWO/Fe/TP1C were carefully analyzed using the Fe K‐edge data obtained from X‐ray absorption spectroscopy. As exhibited in Figure 2g, the extended X‐ray absorption fine structure (EXAFS) in r‐space, with a Fourier transformation, revealed a prominent shell peak at ≈1.5 Å for BWO/Fe/TP1C, which can be attributed to the Fe─O/N bonding. Additionally, wavelet transforms (WT) of k‐space were performed to differentiate the atomic environments, which also proved the Fe─O/N coordination bond in the BWO/Fe/TP1C (Figure 2h). As illustrated in Figure 2i, the X‐ray absorption near edge structure (XANES) spectra further indicated that Fe^3+^ was successfully anchored on the skeleton of COFs.

**Figure 3:**
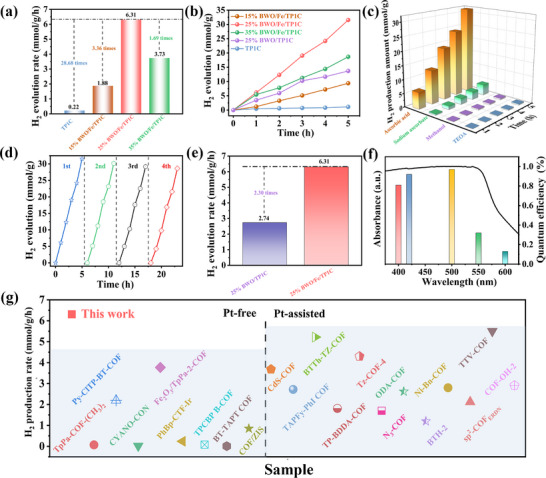
a,b) Photocatalytic performance for synthesized materials. c) The photocatalytic H_2_ production activity of 25% BWO/Fe/TP1C in different sacrificial agents. d) The cyclic experiments of 25% BWO/Fe/TP1C. e) Comparison of photocatalytic competence between 25% BWO/TP1C and 25% BWO/Fe/TP1C. f) The apparent quantum efficiency (AQE) for 25% BWO/Fe/TP1C. g) Comparison of photocatalytic activity between 25% BWO/Fe/TP1C and reported photocatalysts.

Subsequently, under visible light, the photocatalytic H_2_ production performance of prepared materials was investigated. The photocatalytic system was maintained at around 5 °C through a precisely controlled water circulation cooling system (Figure S1, Supporting Information). Screening of different sacrificial agents revealed that L‐Ascorbic acid enabled optimal hydrogen evolution performance for the BWO/Fe/TP1C composite, as demonstrated by the comparative activity data presented in **Figure** **3**c. Therefore, L‐Ascorbic acid can be served as a suitable hole scavenger to assist prepared samples in photocatalytic hydrogen production. It is shown in Figure 3a that bare TP1C exhibited poor photocatalytic H_2_ evolution performance, which can be attributed to the significant photo‐induced carrier recombination. When COFs were combined with BWO, the photocatalytic activity of the materials was significantly enhanced. Moreover, the photocatalytic hydrogen production rate of the as‐prepared 25% BWO/Fe/TP1C was further improved after the introduction of Fe ions on the skeleton of COFs, which was ≈6.31 mmol g^−1^ h^−1^, being 2.3 times as high as that of 25% BWO/TP1C (Figure 3e). In addition, control experiments showed that the free Fe ions in the solution had very limited improvement on the photocatalytic activity of 25% BWO/TP1C (Figure S30, Supporting Information). Clearly, as the proportion of BWO in the composites was increased, the photocatalytic activity progressively improved. Nonetheless, excessive BWO loading was found to negatively impact TP1C's light absorption characteristics, consequently reducing the composite's photocatalytic hydrogen generation performance. The optimal 25% BWO/Fe/TP1C formulation demonstrated a hydrogen evolution rate of 31.55 mmol g^−1^ during 5 h of irradiation without co‐catalysts (Figure 3b). Furthermore, this composite exhibited an apparent quantum efficiency (AQE) of 0.92% at 420 nm illumination (Figure 3f). Apparently, 25% BWO/Fe/TP1C owned a high photocatalytic hydrogen production activity that exceeded a host of the reported photocatalysts without any co‐catalysts, and even surpassed many photocatalysts that used Pt as co‐catalysts (Figure 3g; Table S6, Supporting Information). Additionally, the durability and recyclability of the as‐synthesized products were assessed by cyclic testing experiments. The photocatalytic hydrogen production activity of 25% BWO/Fe/TP1C almost remained unchanged after the fourth cycle test, proving the recyclability and sustainability of the photocatalysts (Figure 3d). Furthermore, the Fourier‐transform infrared (FT‐IR) spectra and SEM images of the 25% BWO/Fe/TP1C composite before and after the photocatalytic reaction were analyzed. The results showed that the peak intensity, position, and morphological structure of BWO/Fe/TP1C also remained unchanged after photocatalytic testing, confirming its excellent stability (Figures S31 and S32, Supporting Information).

**Figure 4:**
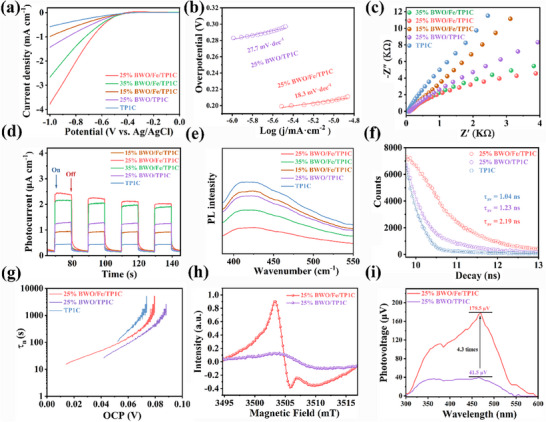
a) LSV measurements for as‐prepared samples. b) The Tafel slopes of 25% BWO/TP1C and 25% BWO/Fe/TP1C. c–e) EIS Nyquist plots, Transient photocurrent curves and PL spectra for synthesized catalysts. f) Time‐resolved PL spectra (λ_ex_ = 375 nm) of prepared materials. g) The carriers' average lifespan. h) EPR spectra of 25% BWO/TP1C and 25% BWO/Fe/TP1C in solid‐state upon light irradiation. i) SPV tests for 25% BWO/TP1C and 25% BWO/Fe/TP1C.

Photoelectrochemical characterization was systematically performed to elucidate the mechanistic origins of the improved photocatalytic performance, with particular focus on evaluating charge carrier separation dynamics and interfacial transfer kinetics. First, the linear sweep voltammetry (LSV) results revealed that 25% BWO/Fe/TP1C exhibited a higher current density in comparison with 25% BWO/TP1C and pure TP1C at the same potential, indicating its enhanced reduction capability for driving the H_2_ evolution reaction (**Figure** **4**a). Compared with 25% BWO/TP1C, 25% BWO/Fe/TP1C exhibited a lower Tafel slope, indicating its stronger carrier separation ability (Figure 4b). As shown in Figure 4c, the electrochemical impedance spectroscopy (EIS) indicated that 25% BWO/Fe/TP1C owned the smallest charge transfer resistance compared with other samples. The carrier separation and transfer performance were also assessed through transient photocurrent measurements (Figure 4d). Compared to bare TP1C and other synthesized composites, 25% BWO/Fe/TP1C hybrid material exhibited the strongest photocurrent response under light excitation, indicating that the formation of heterojunction effectively promote the separation and migration of photo‐induced carriers.

The separation and dynamics of the photogenerated charges were further investigated using photoluminescence (PL) and time‐resolved photoluminescence (TRPL) spectroscopy. As shown in Figure 4e, TP1C and 25% BWO/TP1C exhibited higher fluorescence intensity due to their higher carrier recombination rate. Notably, Fe ions incorporation induced substantial fluorescence quenching in the 25% BWO/Fe/TP1C composite, indicative of enhanced electron transfer kinetics.^[^ ^39^ ^]^ In addition, TRPL spectroscopy quantitatively verified improved charge separation in the 25% BWO/Fe/TP1C composite, exhibiting an extended carrier lifetime (2.19 ns) compared to its Fe‐free counterpart (1.23 ns) (Figure 4f). Furthermore, based on the open circuit voltage decay (OCVD) experiments (Figure S33, Supporting Information), the carrier lifetime of the 25% BWO/Fe/TP1C composite was also longer than that of 25% BWO/TP1C, indicating a reduced carrier recombination rate (Figure 4g), which was in agreement with the results obtained from TRPL measurements.^[^ ^40^ ^]^ Additionally, 25% BWO/Fe/TP1C exhibited a stronger electron paramagnetic resonance (EPR) signal than 25% BWO/TP1C, indicating that BWO/Fe/TP1C could generate more free electrons to participate in the photocatalytic reaction (Figure 4h).^[^ ^41^, ^42^ ^]^

As shown in Figure 4i, a surface photovoltage (SPV) of ≈41.5 µV was measured in BWO/TP1C, which was attributed to the internal electric field (IEF) generated by the Z‐scheme heterojunction formed between BWO and TP1C. Compared with BWO/TP1C, the SPV of BWO/Fe/TP1C was enhanced by ≈4.3 times because the immobilized Fe ions serve as electron transfer mediators, accelerating charge migration between BWO and TP1C. These findings collectively confirm that the established 2D/2D heterojunction can effectively suppress the recombination of electron‐hole pairs. Especially, when Fe ions were introduced, the transfer and separation ability of charge carriers in hybrid materials was further improved.

Moreover, surface wettability analysis via water contact angle measurements revealed that BWO/Fe/TP1C displayed stronger hydrophilicity than that of BWO/TP1C and pure TP1C. The improved surface wetting property is believed to promote effective reactant enrichment at the catalyst interface, consequently optimizing the photocatalytic water splitting efficiency for hydrogen generation (Figures S34–S37, Supporting Information).

**Figure 5:**
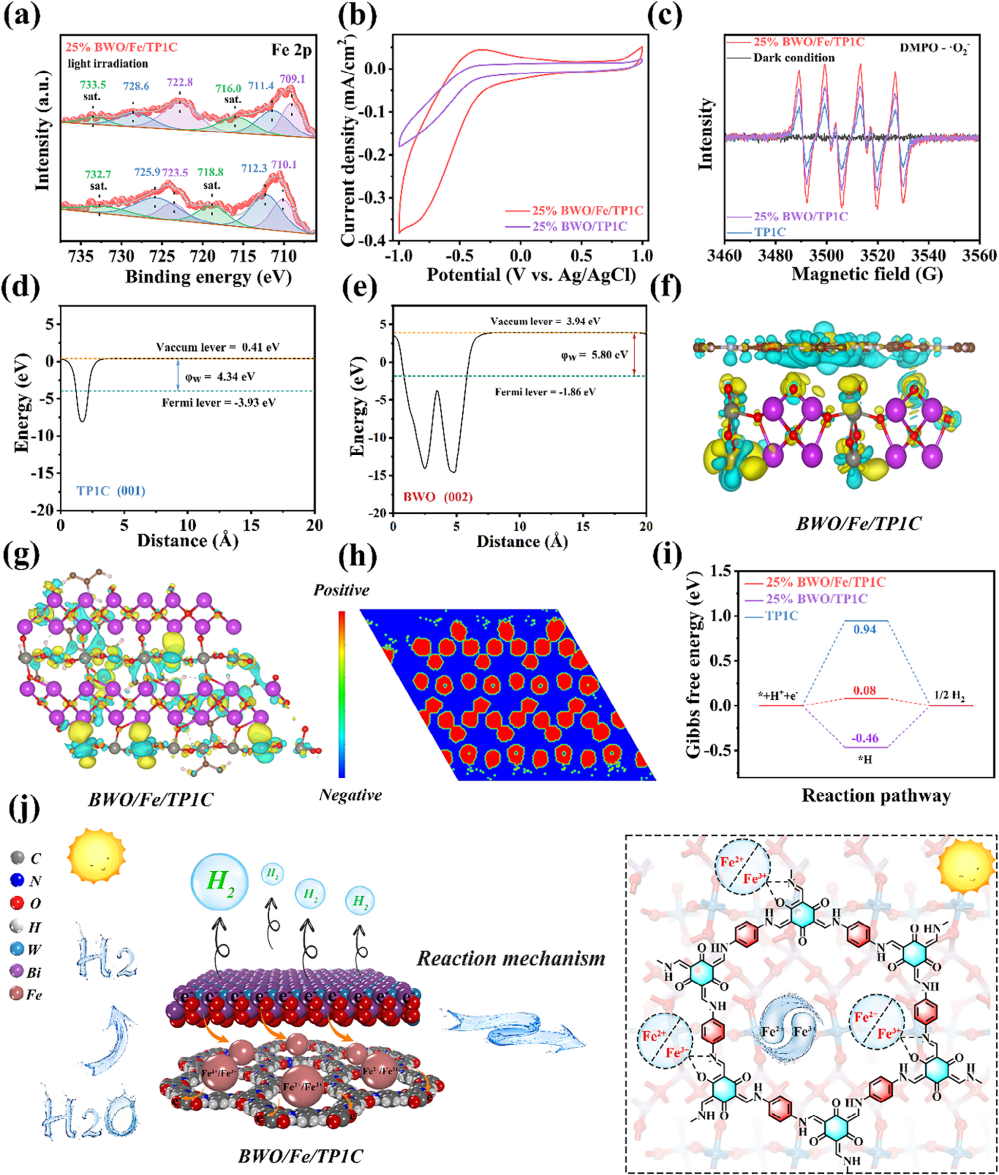
a) In situ XPS spectra of Fe 2p for 25% BWO/Fe/TP1C. b) CV tests for 25% BWO/TP1C and 25% BWO/Fe/TP1C. c) EPR tests for BWO, TP1C and 25% BWO/Fe/TP1C. d,e) Work functions of BWO and TP1C by DFT method. f,g) The calculated charge density difference within BWO/Fe/TP1C composite. h) The longitudinal section depiction of BWO/Fe/TP1C in figure 5g. i) Gibbs free energy change of hydrogen evolution reaction of prepared samples. j) The photocatalytic hydrogen production mechanism of 25% BWO/Fe/TP1C.

The photocatalytic mechanism of synthesized samples was further explored through in situ XPS tests, cyclic voltammograms (CV) measurements, free radical capture experiments and Density Functional Theory (DFT) calculations. As shown in **Figure** **5**a, the XPS peaks of BWO/Fe/TP1C at 710.9 and 724.3 eV referred to Fe 2p_3/2_ and Fe 2p_1/2_, respectively. The Fe 2p spectrum can be classified as six peaks, in which the two fitted peaks at 723.5 and 710.1 eV were attributed to presence of Fe^2+^, and the two peaks at 725.9 and 712.3 eV were associated with existence of Fe^3+^. Furthermore, the remaining two peaks at 732.7 and 718.8 eV were identified as satellite peaks. Besides, under light illumination, the peak area belonging to Fe^2+^ increased, while the peak area belonging to Fe^3+^ decreased, and the binding energy shifted toward a decreasing direction, which was attributed to the partial reduction of Fe^3+^ to Fe^2+^ by photogenerated electrons under light irradiation. Moreover, it can be analyzed from the CV tests that BWO/Fe/TP1C showed quasi‐reversible redox peaks at ≈−0.36 and −0.81 V (vs Ag/AgCl), respectively, which were associated with the transformation between Fe^3+^ and Fe^2+^ (Figure 5b). The presence of Fe^3+^ and Fe^2+^ suggested that there was an interfacial charge transfer in the BWO/Fe/TP1C heterojunction, and that the cycling of Fe^3+^/Fe^2+^ accelerated the migration and separation of the carriers. Additionally, as depicted in Figure S15 (Supporting Information), the W 4f peak of BWO/Fe/TP1C shifted toward a decreasing direction compared to that of BWO, whereas, under light irradiation, the peak position underwent an upshift.

Similarly, the Bi 4f peak of BWO/Fe/TP1C experimented a downshift in comparison with that of pure BWO, while it moved in the opposite direction under illumination conditions (Figure S16, Supporting Information), which indicated that under light illumination, electrons migrated from BWO to TP1C. The results of in situ XPS and CV experiments demonstrated the cycling of Fe^3+^/Fe^2+^, which played an important role in accelerating the transfer of charges. Moreover, under light irradiation, the electrons in the BWO/Fe/TP1C composites were transferred from BWO to TP1C, thus confirming the Z‐scheme charge transfer mechanism.

Afterward, the reactive oxygen species produced during photocatalysis were characterized using EPR measurements, employing 5,5‐Dimethyl‐1‐pyrroline N‐oxide (DMPO) as a spin trap for superoxide radicals (∙O_2_^−^). When exposed to visible light irradiation, distinct EPR signals attributed to DMPO‐∙O_2_^−^ complexes were detected in TP1C, BWO/TP1C and BWO/Fe/TP1C composite. Notably, the signal intensity from the BWO/TP1C was significantly stronger than that from TP1C (Figure 5c). It can be analyzed from the energy band structure that the CB potential of bare BWO was ≈0.31 V, which was more positive than the reduction potential of O_2_/∙O_2_^−^ (−0.33 V vs NHE) (Figure S21, Supporting Information). In contrast, TP1C, with stronger reduction activity, can effectively convert O_2_ to ∙O_2_^−^. If an II‐scheme heterojunction was formed between BWO and TP1C, the photo‐induced electrons from TP1C would migrate to BWO, leading to a reduced reduction activity in BWO, which prevented the production of ∙O_2_^−^. Accordingly, the DMPO‐∙O_2_^−^ signals from BWO/TP1C should be weaker than those from TP1C. However, the in situ EPR analysis revealed an inverse correlation, indicating that the BWO/TP1C composites likely operated through a Z‐scheme electron transfer pathway. The electrons from BWO tend to recombine with the holes in TP1C, leaving the electrons in TP1C and the holes in BWO. As a result, TP1C maintained strong reduction activity due to the presence of the localized electrons, while BWO preserved significant oxidation activity due to the localized holes, leading to a stronger photocatalytic performance. In addition, BWO/Fe/TP1C exhibited a stronger DMPO‐∙O_2_^−^ signals compared to BWO/TP1C because the introduction of Fe ions accelerated the electron migration from BWO to TP1C, which resulted in a greater accumulation of photogenerated carriers, facilitating more efficient oxygen reduction to generate ∙O_2_^−^.

To gain deeper insights into the interfacial charge migration behavior, DFT simulations were systematically performed on the constructed BWO/Fe/TP1C system. Based on the DFT calculation results, the work function of the (002) crystal plane of BWO was 5.80 eV, which was higher than that of the (001) crystal plane of TP1C (4.34 eV) (Figure 5d,e). The disparity in work functions between the two materials drove electron transfer from TP1C to BWO until E_f_ equilibrium was reached, resulting in band edge bending and IEF.^[^ ^43^, ^44^ ^]^ A 3D variation in charge density was achieved by BWO/Fe/TP1C hybrid materials (Figure 5f,g). The yellow and cyan regions signified charge accumulation and depletion, respectively. Notably, the BWO surface predominantly displayed yellow regions, interspersed with some cyan areas. Conversely, TP1C surface predominantly exhibited cyan coloration with sporadic yellow zones, as revealed by charge density difference mapping. Quantitative interfacial analysis demonstrated significant electron migration from the TP1C framework to BWO at their junction.^[^ ^45^ ^]^ This directional charge transfer was particularly evident in the cross‐sectional charge density distribution profile (Figure 5h), which unambiguously confirmed the spontaneous electron donation from TP1C to BWO under dark conditions, effectively validating the Z‐scheme charge transfer pathway within the BWO/Fe/TP1C heterojunction. Besides, the free energies of H adsorption (ΔG_H*_) for prepared samples was calculated by DFT method. Obviously, 25% BWO/Fe/TP1C displayed weakened ΔG_H*_ value compared with 25% BWO/TP1C and bare COFs, promoting the subsequent desorption of H_ads_ and enhancing the rapid H_2_ production rate (Figure 5i), which also reflected the important role of Fe ions in accelerating charge transfer. Furthermore, both the BWO and TP1C displayed a 2D structure, enabling close contact, strong interaction and an optimized electronic architecture. The distinctive Z‐scheme charge transfer pathway, enabled by the 2D mesoporous framework, generated an IEF that markedly enhanced the directional migration of photogenerated carriers while suppressing their recombination. This optimized charge transport mechanism resulted in substantially improved photocatalytic activity. Comprehensive characterization through the EPR experiments, in situ XPS tests and DFT simulations collectively verified the critical function of Fe coordination and the Z‐scheme charge transfer system in the BWO/Fe/TP1C hybrid material.

Integrating the comprehensive experimental findings with DFT calculation results, the photocatalytic mechanism of the fabricated Z‐scheme heterojunction composed of 2D materials can be thoroughly confirmed (Figure 5j). In the hybridized material, BWO and TP1C were in close contact and electrons flowed from TP1C to BWO, driving the E_f_ toward equilibrium at the interface. Consequently, an IEF was formed between TP1C and BWO. Under the influence of the IEF, the energy bands of the material were bent, and the photogenerated electrons of BWO reduce Fe^3+^ to Fe^2+^, which subsequently bound to the photogenerated holes of TP1C, while Fe^2+^ was oxidized to Fe^3+^. Simultaneously, the electrons retained in TP1C's LUMO actively participated in proton reduction due to their favorable thermodynamic driving force for hydrogen evolution. The holes retained in the VB of BWO, with strong oxidation capability, were consumed by L‐Ascorbic acid. The formation of Z‐scheme heterojunctions not only suppressed the recombination of charge carriers, but also endowed integrated materials with strong redox properties. It is worth noting that Fe ions were firmly fixed on the skeleton of COFs, which can maintain the stability and activation state for a long time. The cycling of Fe^3+^/Fe^2+^ accelerated the transmission of charges, further improving the separation efficiency of charge carriers. Besides, from a structural engineering standpoint, the 2D porous heterojunction architecture provides multiple synergistic benefits: 1) well‐defined crystalline frameworks enabling directional charge transport, 2) enhanced surface accessibility for reactant adsorption and catalytic site exposure, 3) maximized interfacial contact facilitating interlayer electronic coupling, and (4) mitigated lattice strain preventing atomic interdiffusion. As a result, leveraging the aforementioned advantages, the Z‐scheme heterojunction based on 2D porous materials with cycling of Fe^3+^/Fe^2+^ exhibited excellent photocatalytic performance for H_2_ production.

## 3 Conclusion

In summary, the solvent thermal method and metal coordination strategy were employed to synthesize BWO/Fe/TP1C hybrid materials. Fe ions were firmly fixed on the skeleton of COFs, ensuring their long‐term stability and activation state. With the cycling of Fe^3+^/Fe^2+^, the charge migration rate was obviously improved, further inhibiting the recombination of charge carriers. Furthermore, in situ XPS measurements, DFT simulations and EPR experiments unambiguously confirmed the Z‐scheme electron transport pathway. This configuration simultaneously enhanced charge carrier separation efficiency while preserving exceptional oxidation‐reduction capabilities. Moreover, the structural congruence between 2D mesoporous frameworks facilitated atomic‐scale interfacial contact via Coulombic attraction forces, which offered more reactive sites while preventing lattice mismatches and interatomic fusion, thereby facilitating efficient charge transfer. As a result, the prepared 2D/2D Z‐scheme heterojunction (25% BWO/Fe/TP1C) exhibited a high photocatalytic hydrogen production rate (6.31 mmol·g^−1^·h^−1^), far exceeding that of pure COFs materials (0.22 mmol·g^−1^·h^−1^) and ≈2.3 times that of 25% BWO/TP1C (2.74 mmol·g^−1^·h^−1^), as well as surpassing most COF‐based photocatalysts. In short, this study opens up new possibilities for designing innovative metal doped Z‐scheme heterojunctions using 2D porous materials, aiming at enhanced photocatalytic performance.

## Conflict of Interest

The authors declare no conflict of interest.

## Supporting information



Supporting Information

## Data Availability

The data that support the findings of this study are available from the corresponding author upon reasonable request.
