# Supplementary material for: Anchoring Redox Mediator on COFs for Efficient Solar to Hydrogen Conversion
Source: Adv Mater. 2025 Aug 27;37(45):e10193. doi: 10.1002/adma.202510193 (PMC12617040; doi:10.1002/adma.202510193)
Supplement: Supplementary file 1 — Supporting Information [file ADMA-37-e10193-s001.docx]

Supporting Information

**Anchoring Redox Mediator on COFs for Efficient Solar to Hydrogen Conversion**

Haijun Hu^1,2†^, Xiaodong Sun*^1†^,Yali Ma^3^, Hui Li^2,4^, Wei Zhang^1^, Hua Fan^5^, Hongwei Huang^6^, Tianyi Ma*^2,4^

^1^ Institute of Clean Energy Chemistry, Key Laboratory for Green Synthesis and Preparative Chemistry of Advanced Materials, College of Chemistry, Liaoning University, Shenyang 110036, P. R. China

^2^ Centre for Atomaterials and Nanomanufacturing (CAN), School of Science, RMIT University, Melbourne, VIC 3000, Australia

^3^ College of Chemical Engineering, Shenyang University of Chemical Technology, Shenyang 110142, P. R. China

^4^ ARC Industrial Transformation Research Hub for Intelligent Energy Efficiency in Future Protected Cropping (E2Crop), Melbourne, VIC 3000, Australia

^5^ Aqualux AU Pty Ltd, 12 Kanangra Cres, Clontarf, NSW 2093, Australia

^6^ Beijing Key Laboratory of Materials Utilization of Nonmetallic Minerals and Solid Wastes, National Laboratory of Mineral Materials, School of Materials Science and Technology, China University of Geosciences, Beijing, 100083, P. R. China

^*^Corresponding author.

*E-mail address:* [sunxiaodong@lnu.edu.cn](mailto:sunxiaodong@lnu.edu.cn); tianyi.ma@rmit.edu.au.

***Keywords:*** covalent organic frameworks, photocatalysis, redox mediator.

**1. Experimental Methods**

***1.1. Materials and reagents***

Hexadecyl trimethyl ammonium Bromide (CTAB) (99%), Bismuth nitrate pentahydrate (Bi(NO_3_)_3_·5H_2_O) (99%) were purchased from Damo Chemical Reagent Co., Ltd. Sodium tungstate dihydrate (Na_2_WO_4_·2H_2_O) (99.5%), p-Phenylenediamine (C_6_H_8_N_2_) (97%) were purchased from Aladdin Biochemical Technology Co., Ltd. Hydroxydiacetyl iron, hydrate (Fe(OH)(CH_3_COO)_2_·H_2_O) was purchased from Shanghai Macklin Biochemical Technology Co., Ltd. Acetic acid (CH_3_COOH) (99.5%), Tetrahydrofuran (THF) (99.5%), Ethanol absolute (ETOH) (99.5%), N,N-Dimethylformamide (DMF) (99.5%) were purchased from Tianjin Fuyu Fine Chemical Co., Ltd. 2,4,6-Trihydroxy-benzene-1,3,5-tricarbaldehyde (C_9_H_6_O_6_) (99%) was purchased from Jilin Zhongke Yanshen Technology Co., Ltd.

***1.2. Synthesis of Bi_2_WO_6_ (BWO)***

BWO was prepared according to the previous article with minor alterations.^[1]^ Firstly, 100 mg (0.27 mmol) of CTAB was dissolved in 80 mL of deionized water, and then 165 mg (0.5 mmol) of Na_2_WO_4_·2H_2_O and 485 mg (1 mmol) of Bi(NO_3_)_3_·5H_2_O were added respectively. After 1 h of magnetic stirring, the mixed solution was transferred to a 100 mL Teffon-lined autoclave with stainless steel case and reacted at 120 °C for 24 h. After cooling to room temperature, the products were collected by centrifugation and washed with deionized water and anhydrous ethanol for 3 times, respectively, and then dried in a vacuum drying oven at 60 °C for 12 h.

***1.3 Synthesis of*** ***TpPa-1-COF (TP1C)***

TP1C was synthesized following a previously reported method with slight modifications.^[2]^ A Pyrex tube containing a finely ground mixture of Tp (21 mg, 0.10 mmol) and Pa (17 mg, 0.16 mmol) was charged with 3 mL of DMF and 0.5 mL of 3M acetic acid. The reaction vessel was immediately immersed in liquid nitrogen (77 K) for flash freezing, followed by three freeze-pump-thaw cycles to ensure complete degassing before being sealed under vacuum. The reaction was conducted at 120 °C for 3 days. The resulting product was isolated by centrifugation and subjected to sequential washing with tetrahydrofuran (THF) and acetone (three times each). After that, the resulting products were soaked in anhydrous acetone for 2 days with six solvent exchanges, and then vacuum-dried at 120 °C for 12 h.

***1.4. Preparation of*** ***Bi_2_WO_6_/******TpPa-1-COF (******BWO/TP1C)***

The synthesis steps of BWO/TP1C were similar to that of bare TP1C, except that different amounts of BWO were added.

***1.5. Preparation of*** ***Bi_2_WO_6_/Fe/TpPa-1-COF (BWO/Fe/TP1C)***

An appropriate amount of hydroxydiacetyl iron was dissolved into a 1% acetic acid solution to yield a ferric acetate solution with a concentration of approximately 0.2 M. Then, 20 mg of BWO/TP1C was added into 5 mL of the prepared ferric acetate solution, and magnetically stirred at 50 °C for 8 h. The resulting material was sequentially washed with deionized water and THF, followed by vacuum drying at 60 °C for 12 h.

***1.6. Preparation of*** ***Fe/Bi_2_WO_6_ (Fe/BWO) or Fe/TpPa-1-COF*** ***(Fe/TP1C)***

The synthesis steps of Fe/BWO or Fe/TP1C were similar to that of BWO/Fe/TP1C, except that BWO/TP1C was replaced by BWO or TP1C.

**2. Characterization**

Scanning electron microscopy (SEM) images were obtained with a Hitachi SU-8010 microscope equipped with an energy-dispersive X-ray spectroscopy (EDS) detector. Transmission electron microscopy (TEM) images were recorded using a JEOL JEM-2100 microscope. Powder X-ray diffraction (PXRD) patterns were collected on a Bruker D8 Advance diffractometer with Cu Kα radiation. The X-ray photoelectron spectroscopy (XPS) spectra were acquired using a Thermo Scientific ESCALAB 250Xi spectrometer equipped with a monochromatic Al Kα (1486.6 eV) X-ray source. The optical absorbance spectra were obtained by the Ultraviolet-visible diffuse reflectance spectroscopy (UV-vis DRS) with BaSO_4_ as the reflectance standard (Shimadzu UV-2550). Thermogravimetric analysis (TGA, METTLER TOLEDO TGA/SDTA851) was performed to analyze the thermal stability of as-prepared samples with a heating rate of 10 °C⋅min^-1^ under N_2_ atmosphere. Electron paramagnetic resonance (EPR) measurements were performed on a Bruker EMXplus-6/1 spectrometer (Germany). The specific surface area and pore size distribution of the prepared samples were analyzed by nitrogen adsorption-desorption isotherms using an ASAP 2420 analyzer (Micromeritics, USA). Fluorescence measurements were carried out with the RF-5301PC (Shimadzu, Japan) fluorescence spectrophotometer (excitation wavelength: 350 nm). The Fourier transformed infrared spectroscopy (FT-IR) was performed on a Nicolet Nexus 670 FT-IR spectrophotometer. Surface photovoltage (SPV) measurements were performed using a CEL-SPS1000 system (China Education Au-light Co., Ltd.). The X-ray absorption fine structure (XAFS) spectra of Fe K-edge were collected in transmission mode on a commercial Laboratory-Based XAFS spectrometer (Table XAFS-500A, Specreation Instruments Co., Ltd.). An X-ray tube was used to generate X-ray, and the voltage and current were set to 20 kV and 20 mA. The Ge (620) spherically bent crystal analyzers with a radius of curvature of 500 mm and the R250 mm Rowland circle were used to provide monochromatized X-ray beam.

**3. Photocatalytic H_2_ production properties**

Photocatalytic H_2_ production experiments were carried out via a full glass automatic on-line trace gas analysis system (Labsolar-6A, Beijing Perfectlight Technology Co., Ltd.). The light source is the 300 W Xenon lamp (wavelength: 320-780 nm, PLS-SXE300**/**300UV, light intensity: 100 mW⋅cm^-2^, Beijing Perfectlight Technology Co., Ltd.). Hydrogen was detected and quantitative analyzed by a GC7900 gas chromatography (Shanghai Tianmei Scientific Instrument Co.) with nitrogen as the carrier gas, and the detection interval was 30 min. The catalyst was placed in a custom-made quartz glass reactor, and the reaction temperature was controlled at 5 °C by condensing water. 10 mg of catalyst was added into 100 mL of deionized water containing 100 mg of L-Ascorbic acid as hole scavenger. After that, the suspension was stirred in a 200 mL customized quartz reactor. Before each photocatalytic reaction, the system was vacuum-treated several times to remove the dissolved air. The whole reaction system was illuminated by the Xe light irradiation with a UV cut-off filter (λ ≥ 420 nm). Moreover, the vertical distance between the xenon lamp and the reactor was maintained at 3 cm for all photocatalytic experiments. In addition, the apparent quantum efficiency (AQE) was measured utilizing a xenon lamp equipped with bandpass filters. Photocatalytic experiments were conducted with 10 mg of catalysts, where the wavelengths of the filters used are 400, 420, 500, 550 and 600 nm, respectively. The specific value of AQE was calculated on the basis of the following formula:

$$AQE\left( H_{2} \right)\%=\frac{2\times N(H_{2})}{N(photons)}\times100\%$$

where N(H_2_) refers to the number of H_2_ molecules produced, and N(photons) stands for the number of photons reaching the surface of the reaction suspension.^[3]^

**4. Photo-electrochemical characterization**

The electrochemical measurement was carried out by the electrochemical workstation with the model of CHI 760. The test was carried out in a three-electrode system, in which the Pt sheet was used as the counter electrode, the Ag/AgCl electrode was used as the reference electrode, and the prepared powder sample was used as the working electrode. In addition, 0.2 M Na_2_SO_4_ was utilized as the electrolyte solution, and a xenon lamp equipped with a filter (λ ≥ 420 nm) was utilized as the visible light source. 10 mg of catalyst sample and 20 μL of Nafion solution were added into 1 mL of anhydrous ethanol solution, sonicated for 30 min to make them evenly mixed, and then scraped onto FTO glass to manufacture the working electrode. The Cyclic voltammograms (CV) tests were carried out at a scanning rate of 100 mV·s^-1^ at room temperature. In addition, the Mott-Schottky tests were conducted to acquire the flat-band potential of the material. In the absence of light, the stable open-circuit voltage of this sample was initially measured, and subsequently tested at varying frequencies (500 and 1000 Hz). On the basis of Mott-Schottky equation:

$$\frac{1}{C^{2}}=\frac{2}{N_{D}e\varepsilon_{0}\varepsilon}(E-E_{fb}-\frac{kT}{e})$$

where C refers to the capacitance of the space charge region, N_D_ stands for the electron carrier density, e is the elemental charge, ε_0_ is the permittivity of a vacuum, ε represents the relative permittivity of the semiconductor, E is the applied potential, E_fb_ is the flat band potential, T is the temperature, and k is the Boltzmann constant. The type of semiconductor material is determined by the positive or negative value of the slope of the linear part of the Mott-Schottky curve: Typically, n-type semiconductors possess a positive slope, while p-type semiconductors have a negative slope.^[4]^

In addition, the mean lifespan of the photogenerated carriers determined through the open-circuit voltage decay technique is obtained from equation blow:

$$\tau_{n}=\frac{K_{B}T}{e}\left( \frac{dOCVD}{dt} \right)^{-1}$$

where K_B_ represents the Boltzmann constant, and the value is 1.38× 10^-23^ J·K^-1^, T refers to temperature (298 K), e means the electric charge (1.60 × 10^-19^ C), and dOCVD/dt denotes the derivative of the OCP transient decay.^[5]^

**5. Computational methods**

Our simulations were performed within the framework of density functional theory (DFT) implemented in the Quantum Espresso package (QE).^[6,7]^ The exchange-correlation energies were described using the generalized gradient approximation (GGA) with the Perdew-Burke-Ernzerhof (PBE) functional.^[8]^ To achieve a balance between computational effort and accuracy, energy cutoff is set to 500 eV for the wave function. The geometry relaxation was performed using BFGS quasi-Newton algorithm until the forces on each atom were less than 0.03 eV/Å and the energy difference of consecutive steps was less than 10^-5^ eV. The Brillouin-zone sampling was conducted using Monkhorst-Pack (MP) grids of special points with the separation of 0.04 Å^-1^ for both structural optimization and electronic structure calculations. A vacuum space of 15 Å in the z-direction was used to keep away the layer from interacting with its periodic images. The van der Waals interactions were taken into account using the DFT-D3 approach.^[9,10]^ To eliminate the spurious electrostatic field generated when a polar slab is modeled with three-dimensional periodic boundary conditions, we applied the dipole-moment correction implemented in QE. The work function was evaluated with the HSE06 functional to ensure quantitative accuracy. The bandgap value was determined as the energy difference between the valence band maximum (VBM) and conduction band minimum (CBM) in the calculated band structure. To match the experimental bandgap, a scissors operator was applied to shift the conduction bands uniformly. The free energy of a gas phase molecule or an adsorbate on the surface was calculated by the equation G = E + ZPE − TS, where E is the total energy, ZPE is the zero-point energy, T is the temperature in kelvin (298.15 K is set here), and S is the entropy.


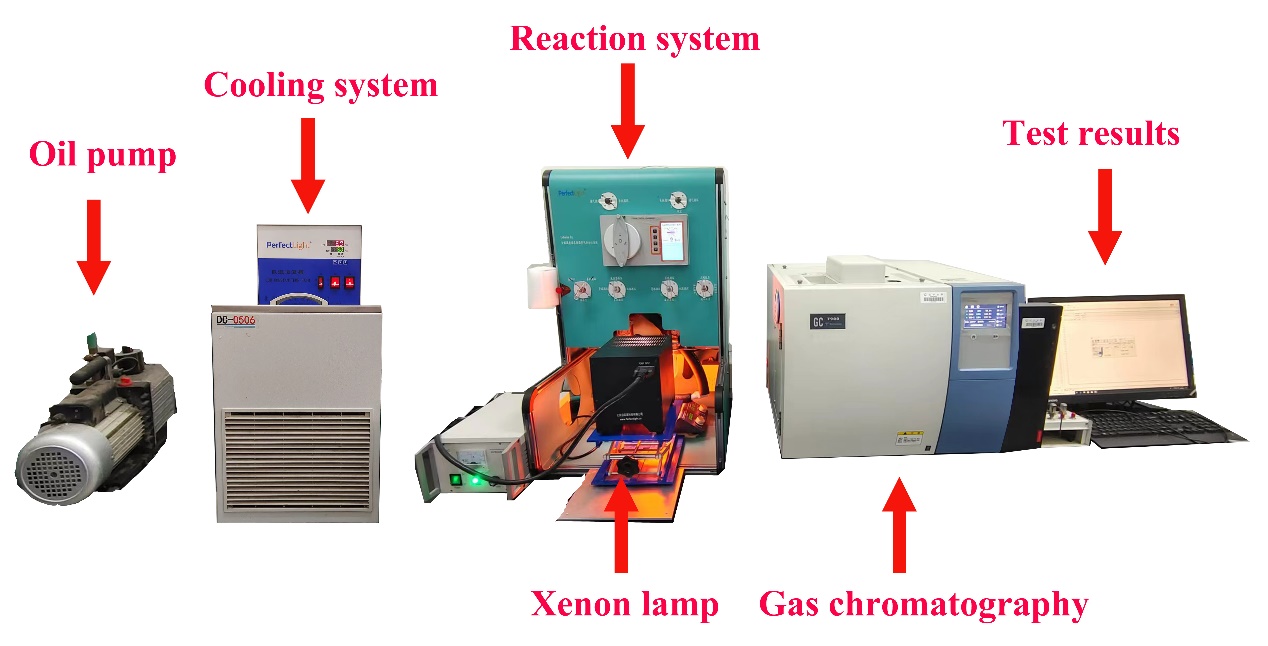


**Fig. S1.** Photocatalytic experimental equipment.

The labsolar-6a all-glass automatic on-line trace gas analysis system, produced by Beijing Perfectlight Technology Co., Ltd., was utilized for conducting the photocatalytic experiments. The illumination was provided by the 300 W Xenon lamp manufactured by Beijing Perfectlight Technology Co., Ltd. Hydrogen was detected and quantitative analyzed by a GC7900 gas chromatography made by Shanghai Tianmei Scientific Instrument Co.


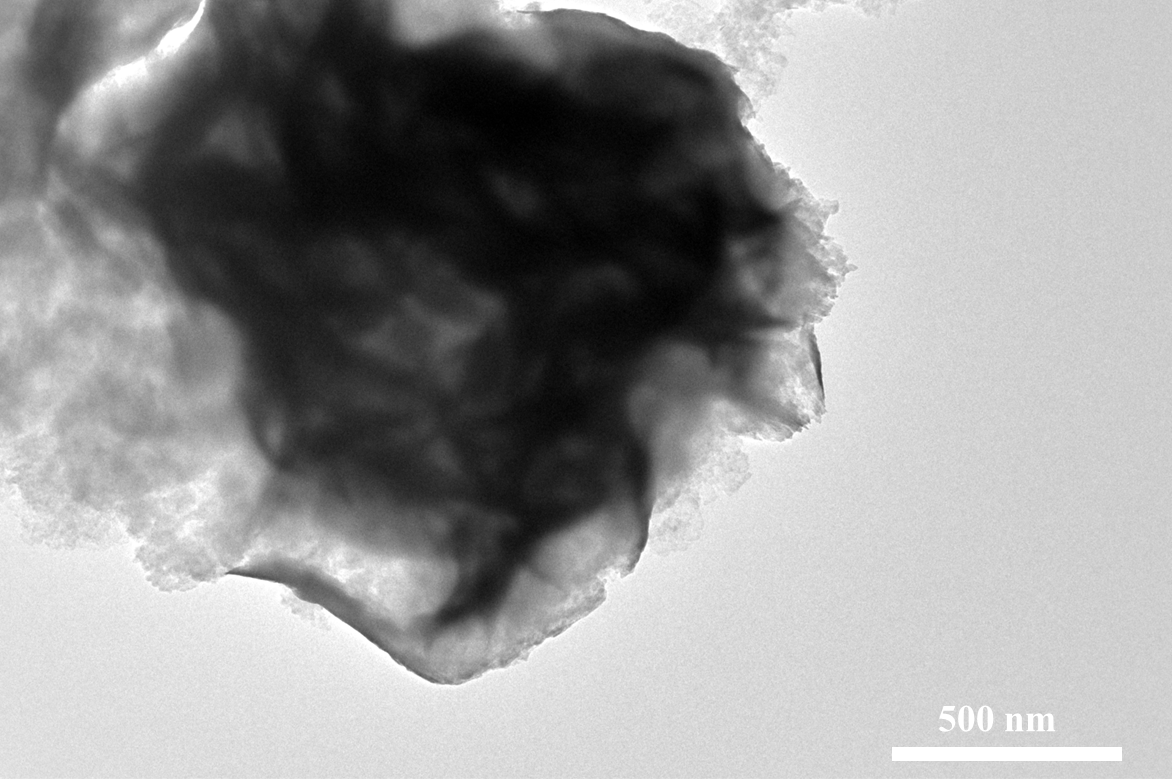


**Fig. S2.** TEM image of BWO.

As shown in Fig. S2, BWO displayed nanosheet structure.


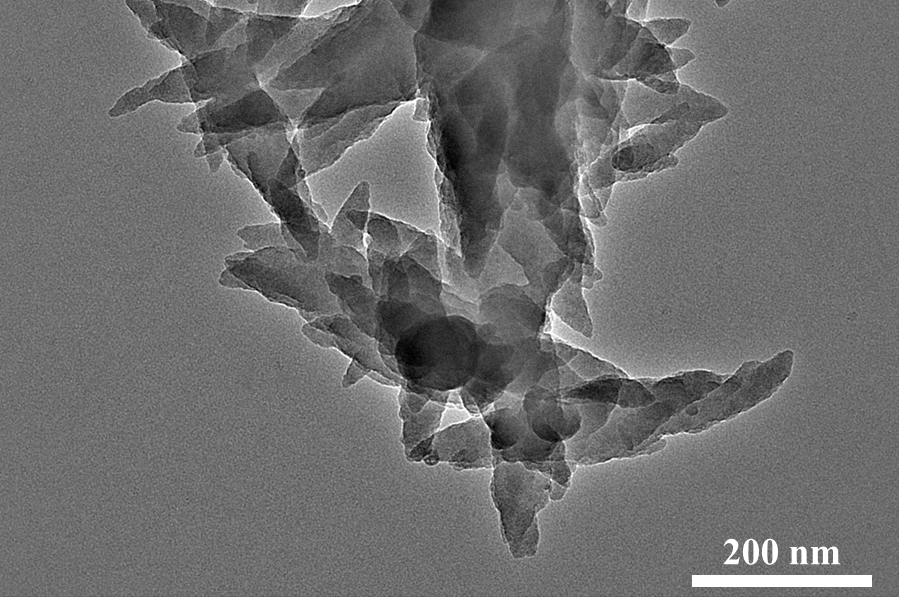


**Fig. S3.** TEM image of TP1C.

As shown in Fig. S3, TP1C exhibited flower-like morphology.


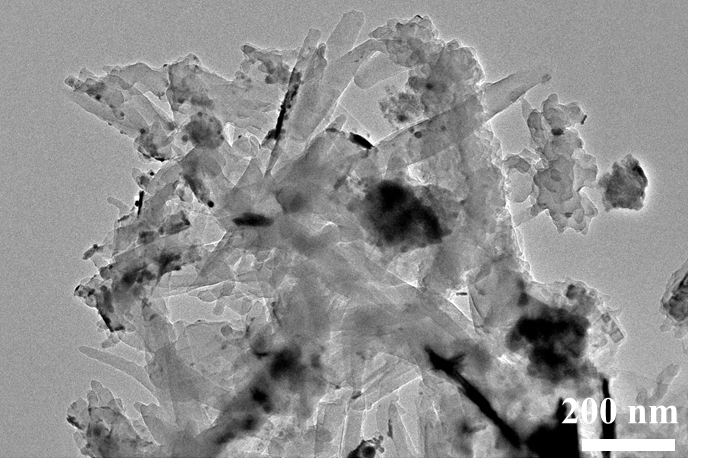


**Fig. S4.** TEM image of BWO/Fe/TP1C.


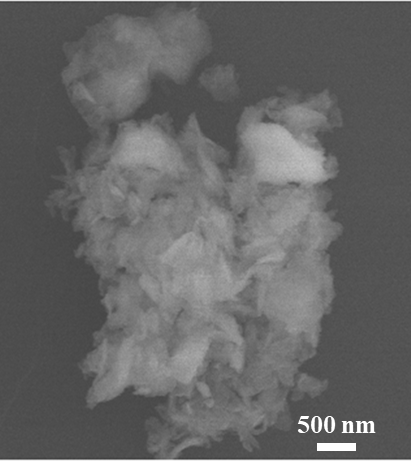


**Fig. S5.** SEM image of BWO/Fe/TP1C.


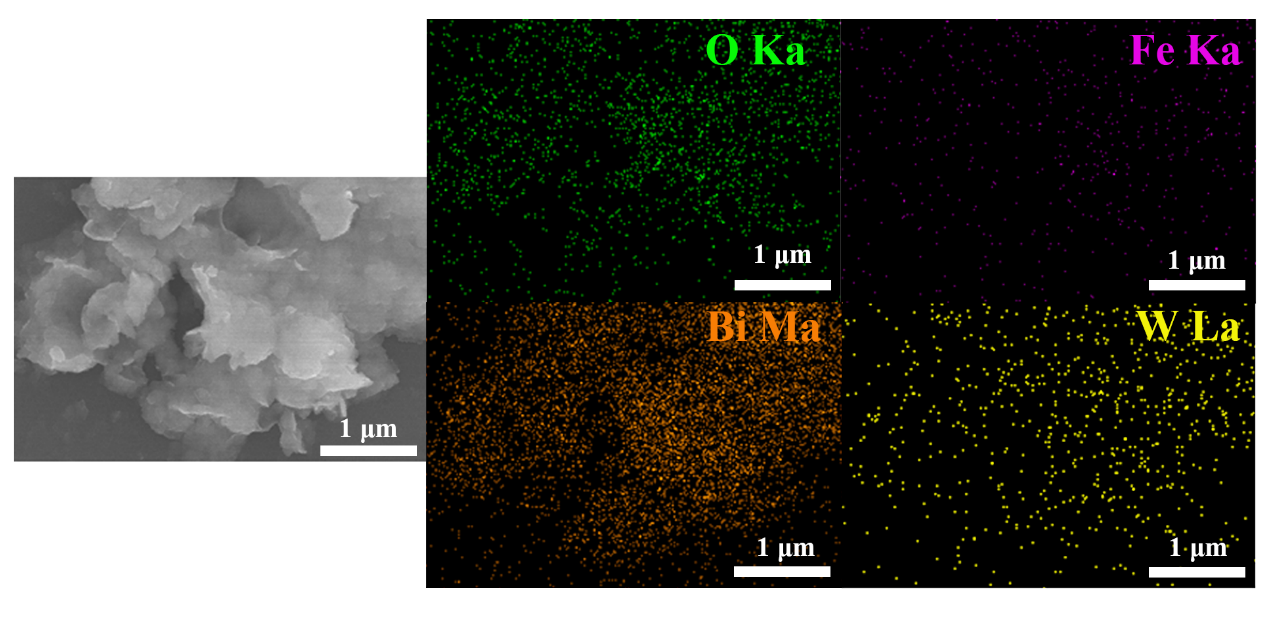


**Fig. S6.** The SEM-EDS pattern of Fe/BWO.

As shown in Fig. S6 and Tab. S1, the Fe content in Fe/BWO was only 0.13%, which can be attributed to the lack of suitable anchoring sites in BWO for effective chemical immobilization of Fe ions.


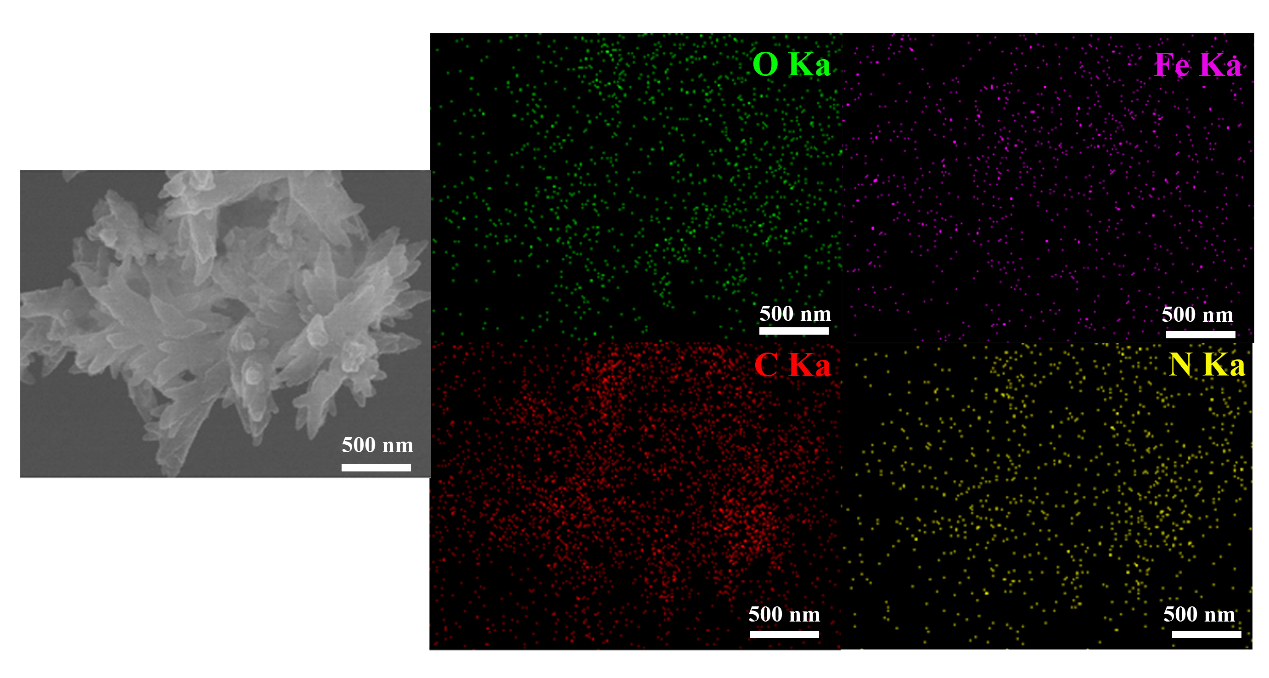


**Fig. S7.** The SEM-EDS pattern of Fe/TP1C.

As shown in Fig. S7 and Tab. S2, Fe/TP1C exhibited a significantly higher Fe content of 5.47% than that of Fe/BWO, providing further evidence for the successful anchoring of Fe ions onto the skeleton of COFs.


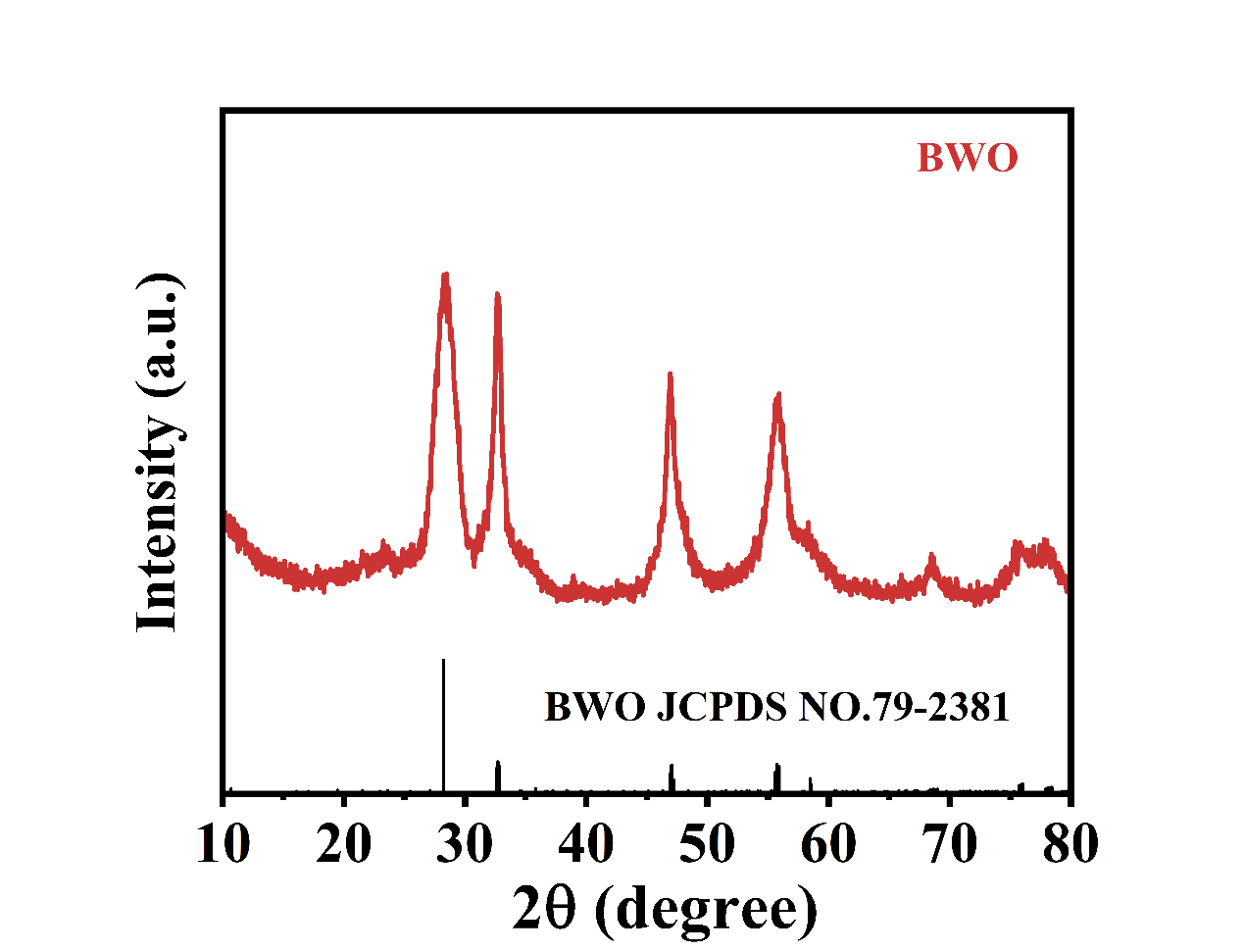
 **Fig. S8.** The PXRD spectra of BWO.

As depicted in Fig. S8, the diffraction peaks of BWO matched well with the standard pattern from JCPDS card (79-2381) for pure BWO, confirming the successful synthesis of BWO nanosheets.


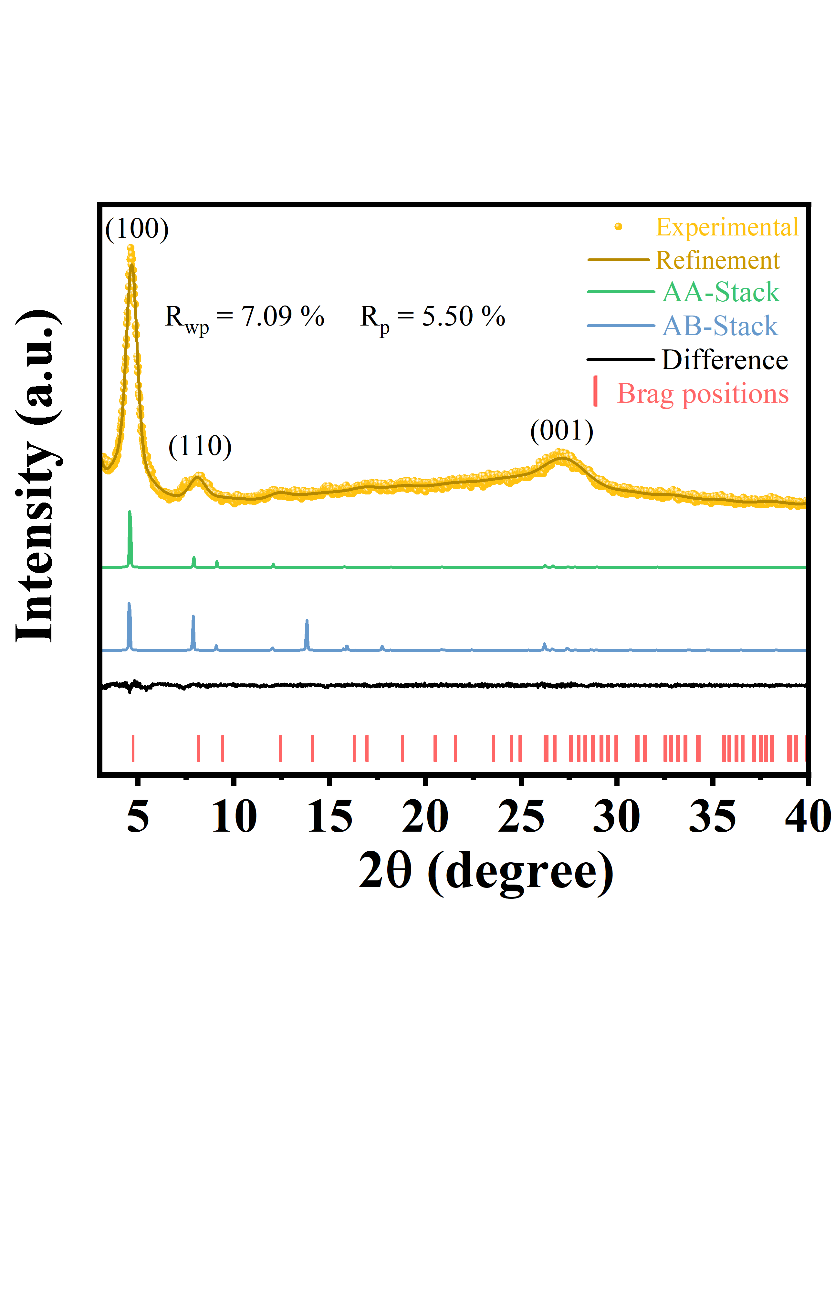


**Fig. S9.** The experimental and simulated PXRD spectra of TP1C.

As shown in Fig. S9, the PXRD peak position of TP1C matched well with the simulated peak, proving that TP1C was successfully prepared. Besides, the Pawley refinement further validated the AA-stacking model for TP1C, with refined parameters (P6/M, a = b = 22.56 Å, c = 3.40 Å, α = β = 90°, γ = 120°) demonstrating excellent agreement with experimental PXRD profiles.


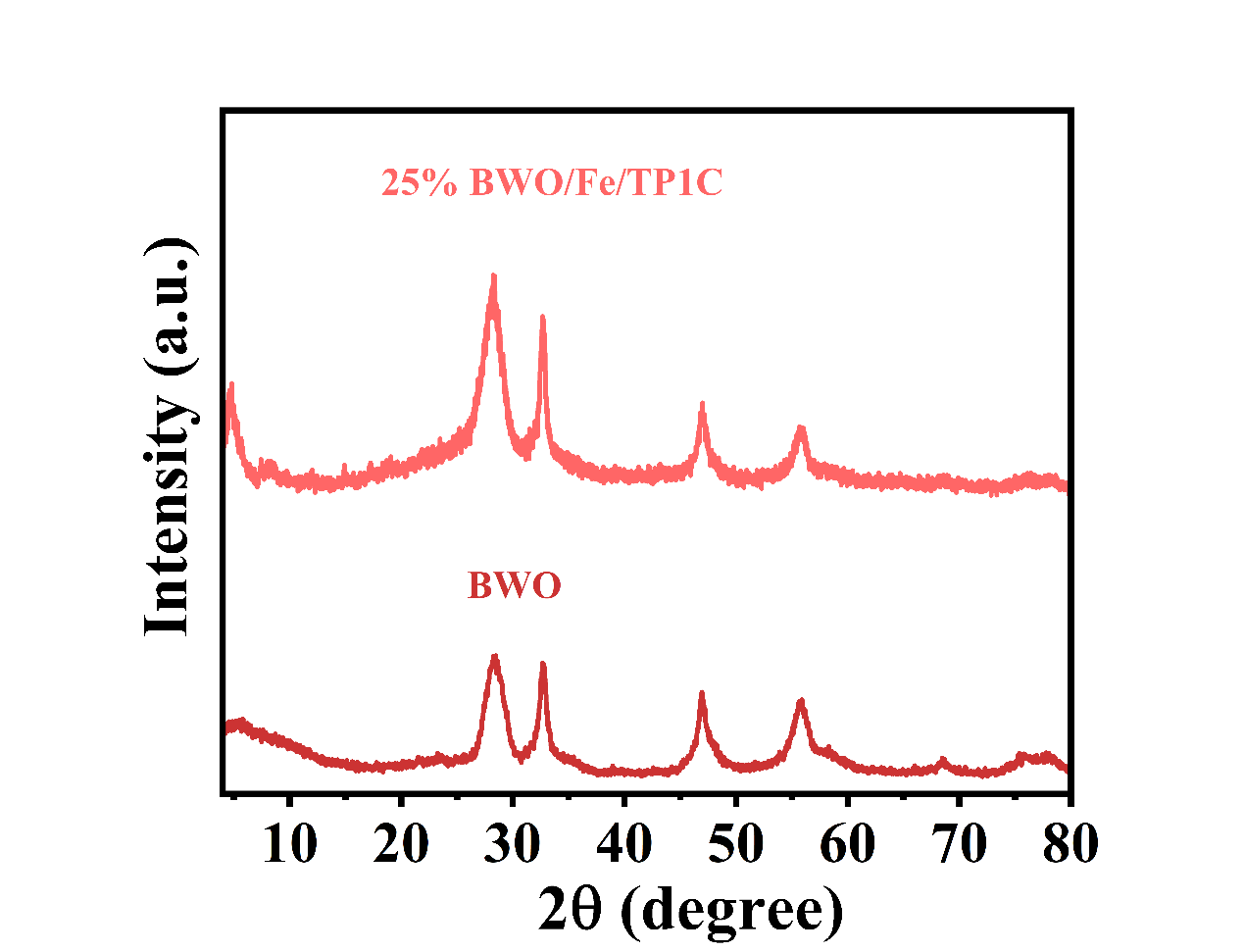


**Fig. S10.** The PXRD spectra of BWO and 25% BWO/Fe/TP1C.

The PXRD pattern of BWO/Fe/TP1C revealed a strong diffraction peak at 4.7°, corresponding to the (100) crystal plane of the TP1C sample, while the other peaks align well with the simulated peaks of BWO, which further indicated that the two materials have been successfully combined and maintained good integrity of the crystal structure during the preparation process.


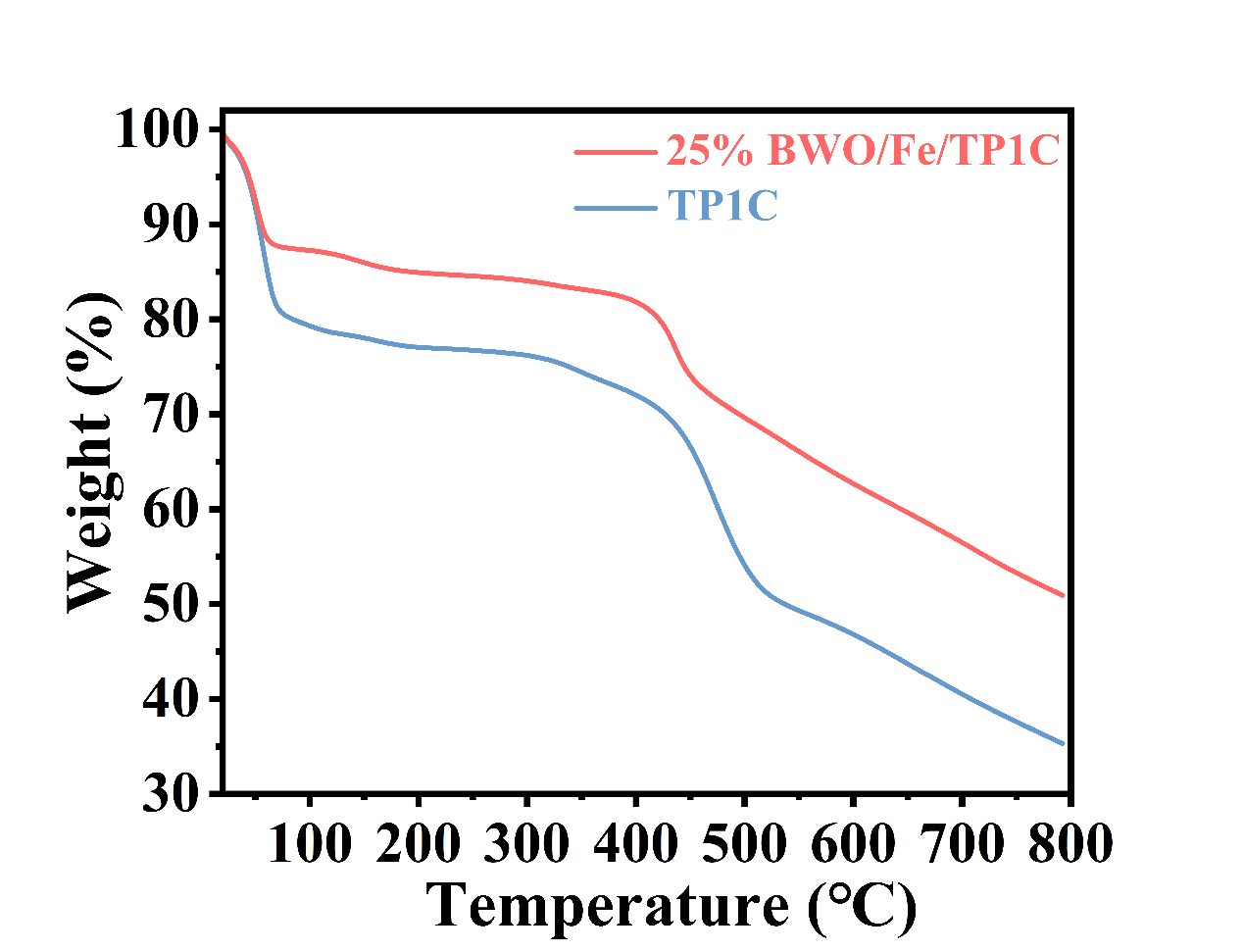


**Fig. S11.** The TG tests for prepared samples.

As shown in Fig. S11, the Thermogravimetric (TG) analysis indicated that the weight of COFs and its hybrid materials decreased as temperature increased. Moreover, BWO/Fe/TP1C and TP1C began to decompose at around 420 ℃ and 450 ℃, demonstrating their good thermal stability.


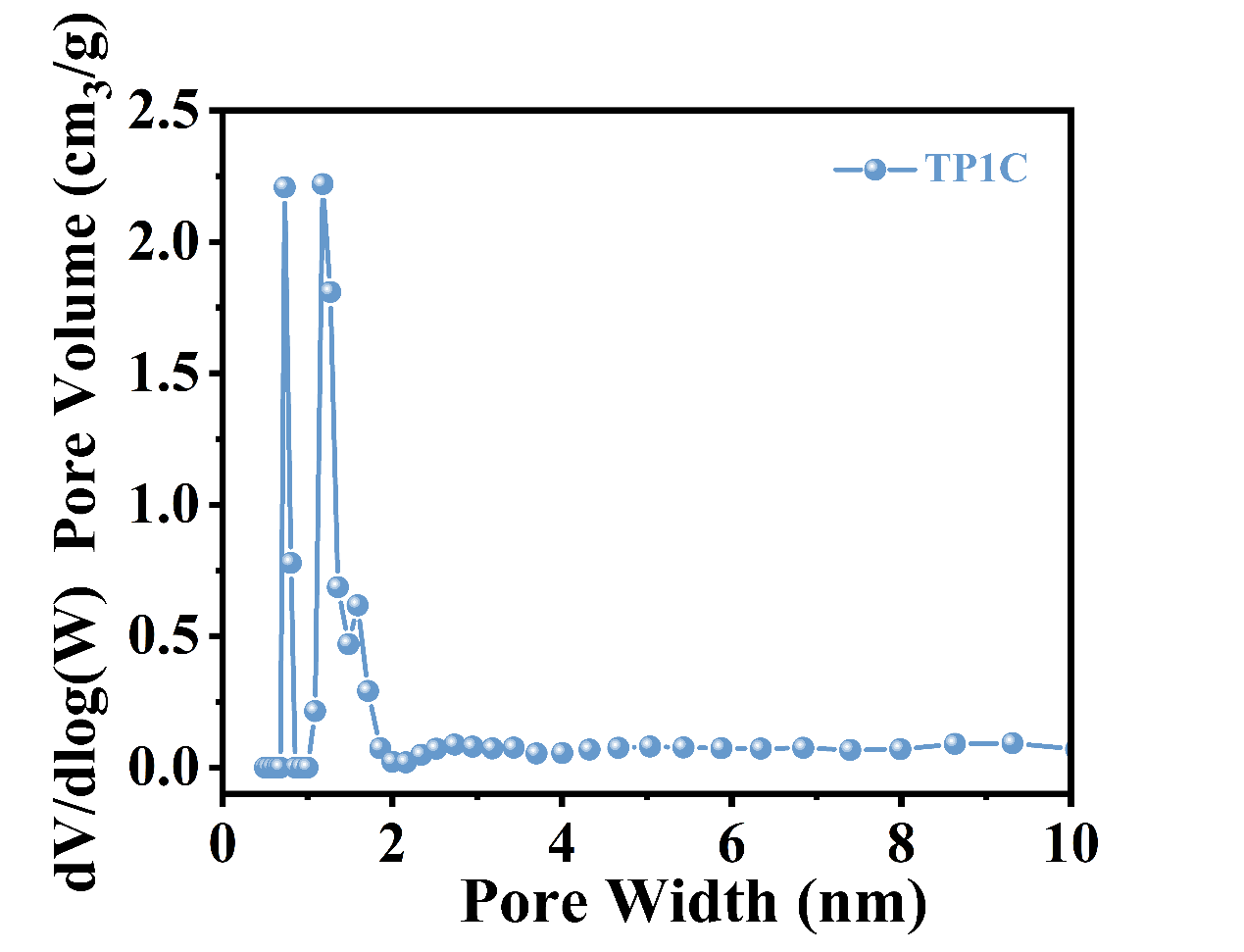
**Fig. S12.** The pore size distribution of TP1C.


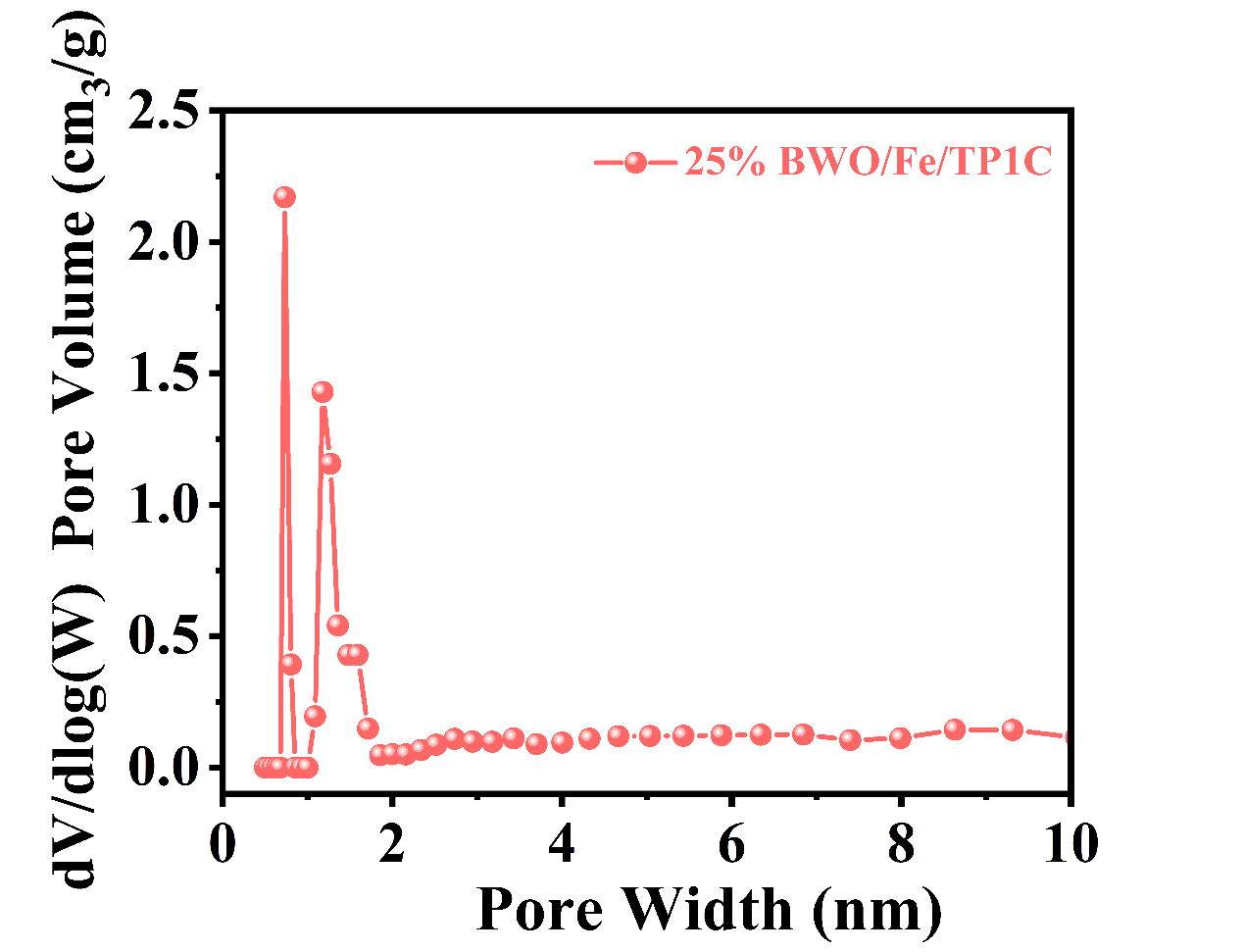


**Fig. S13.** The pore size distribution of 25% BWO/Fe/TP1C.


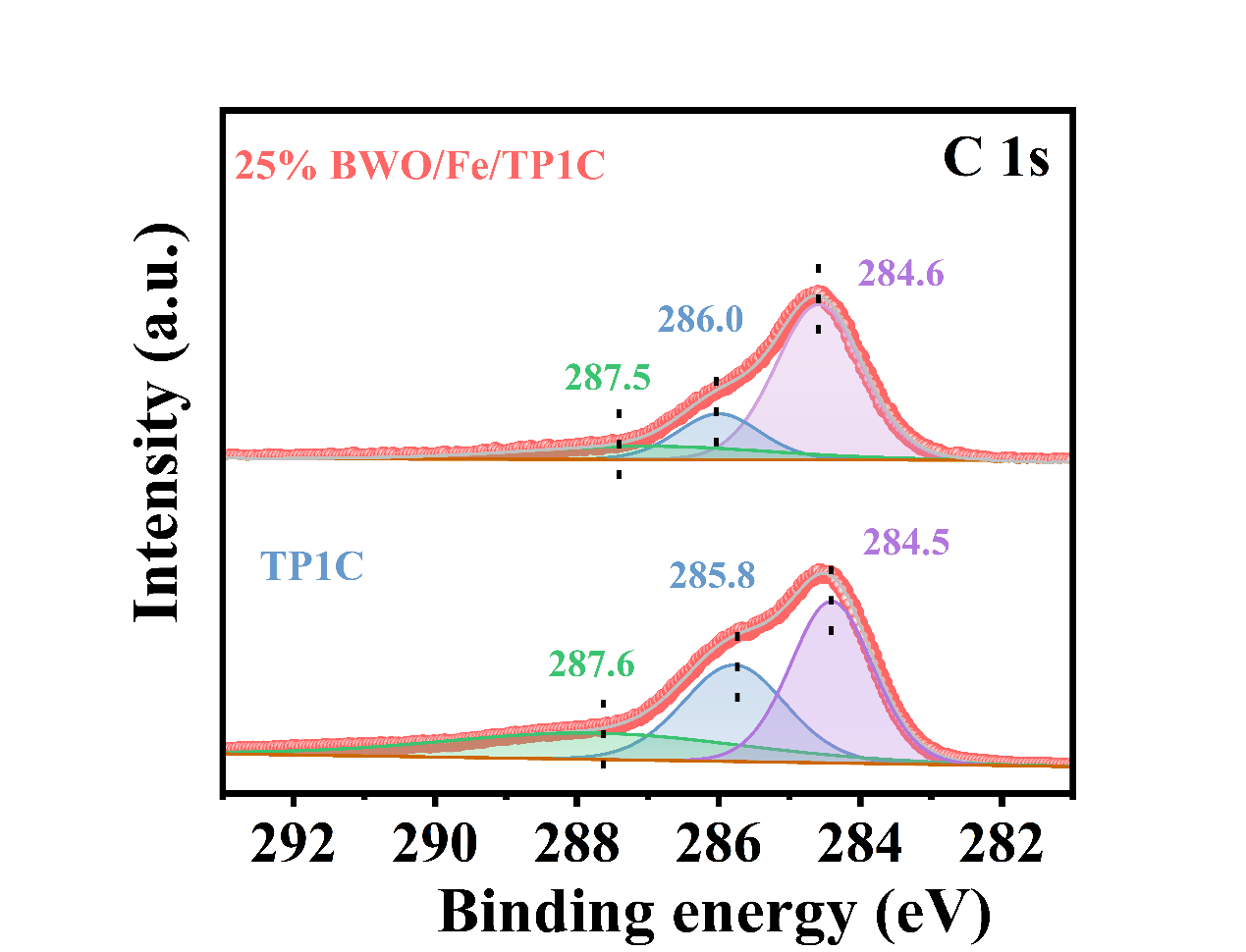


**Fig. S14.** The high-revolution C 1s XPS spectra of prepared samples.

Fig. S14 exhibited the high-resolution C 1s spectrum of prepared materials, which can be divided into the three peaks at 284.6, 286.0 and 287.5 eV for BWO/Fe/TP1C, corresponding to the C=C, C-N and C=O groups, respectively.


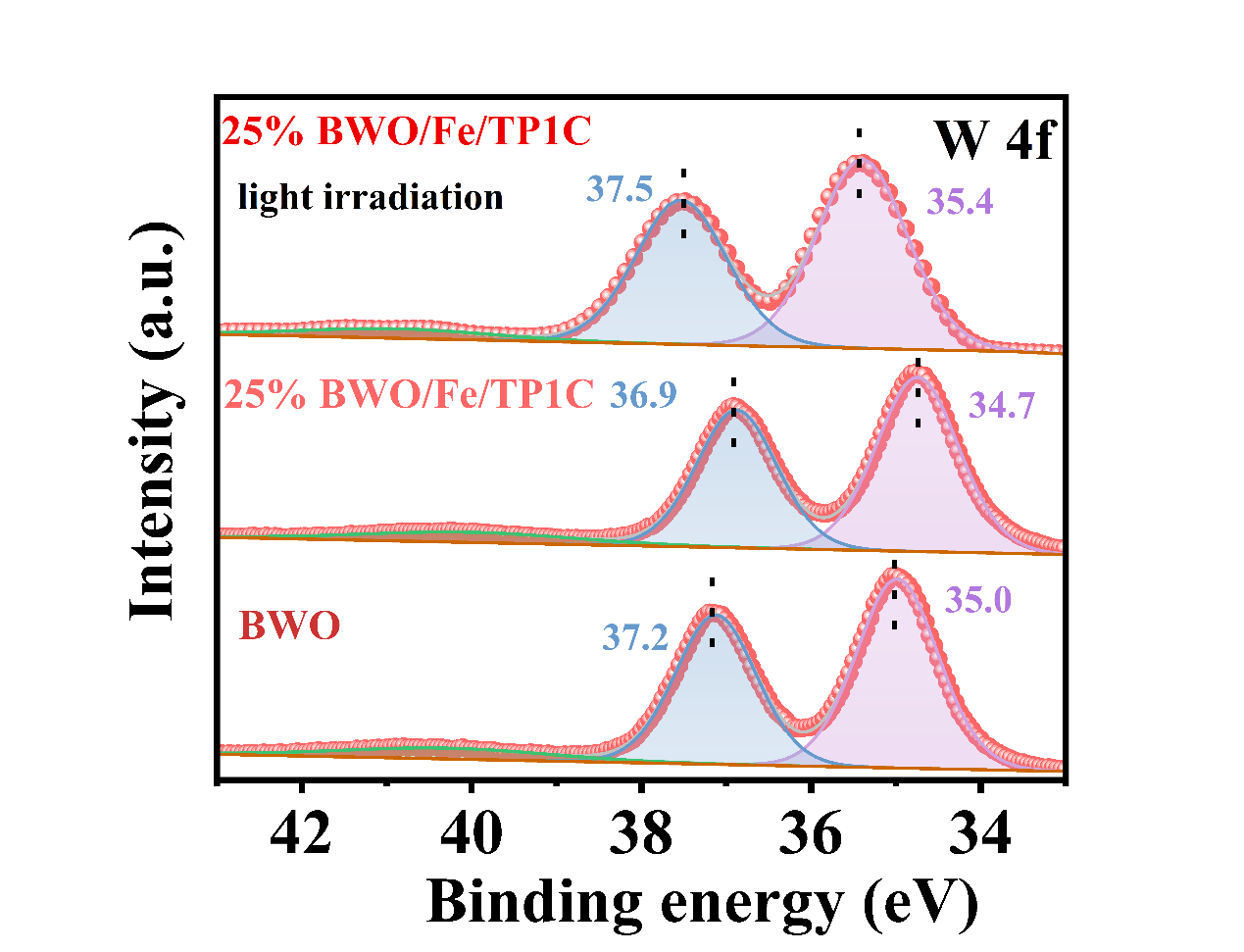


**Fig. S15**. The high-revolution W 4f XPS spectra.

The W 4f XPS spectra of BWO displayed two peaks at 35.0 and 37.2 eV, which stood for W 4f_7/2_ and W 4f_5/2_, proving the presence of W^6+^ in BWO.


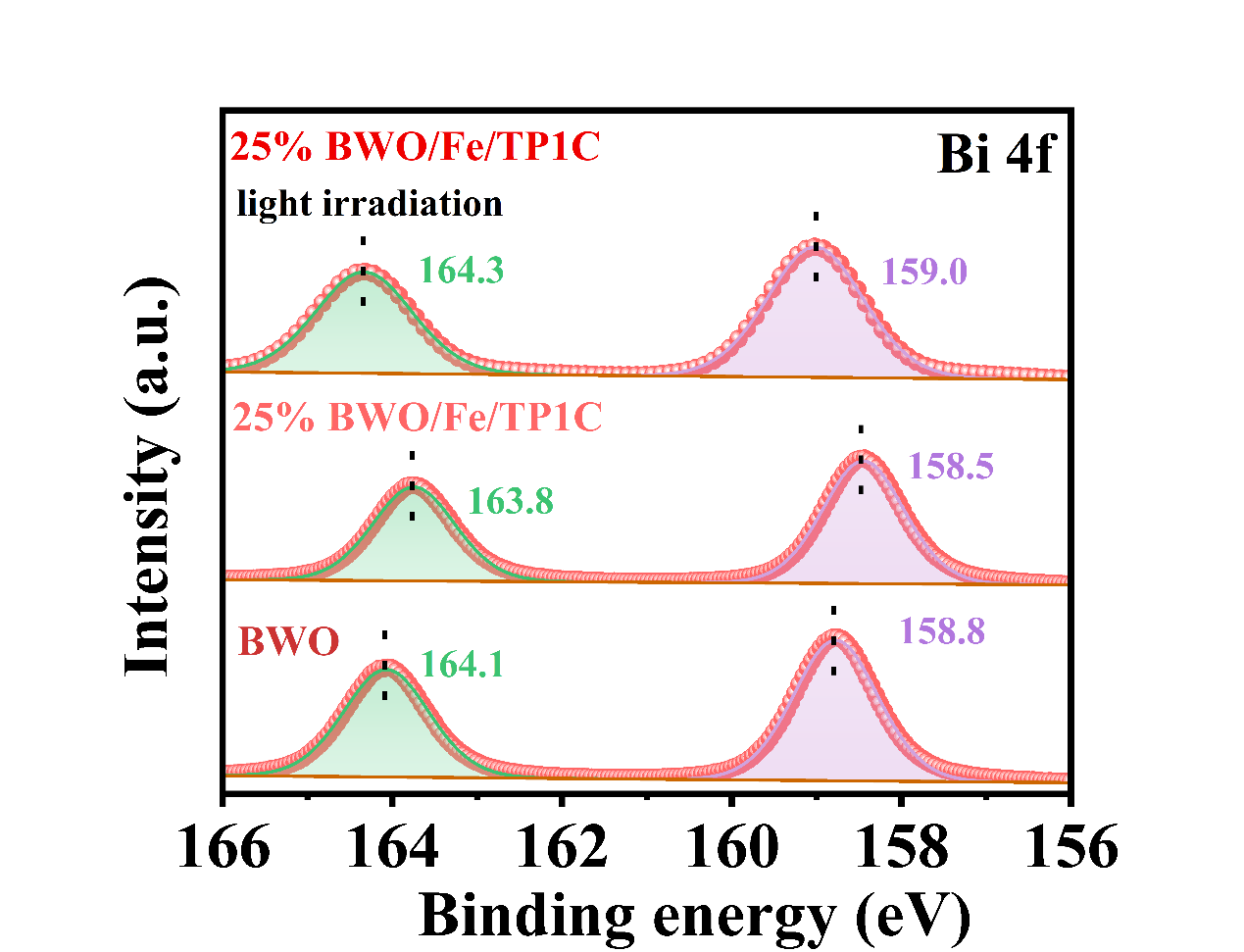


**Fig. S16.** The high-revolution Bi 4f XPS spectra.

Fig. S16 showed Bi 4f spectra of BWO, displaying two peaks at 158.8 and 164.1 eV, respectively. These binding energy peaks were referred to Bi 4f_7/2_ and Bi 4f_5/2_, indicating the presence of Bi^3+^.


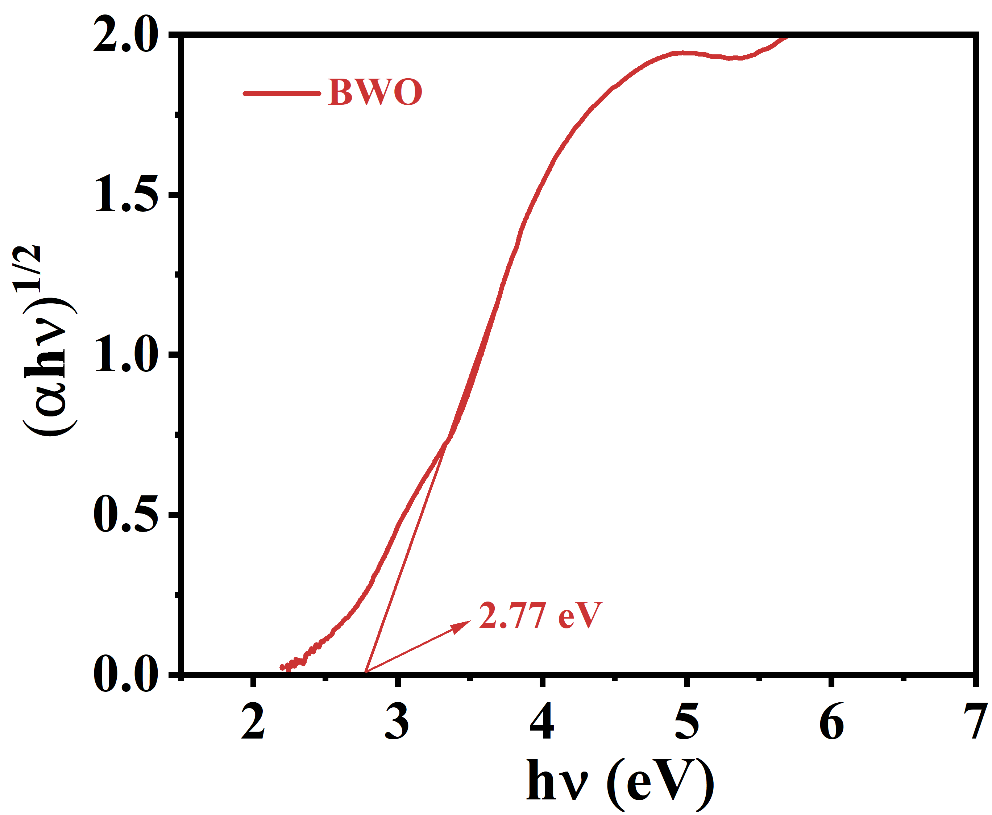


**Fig. S17.** Tauc plots of BWO.


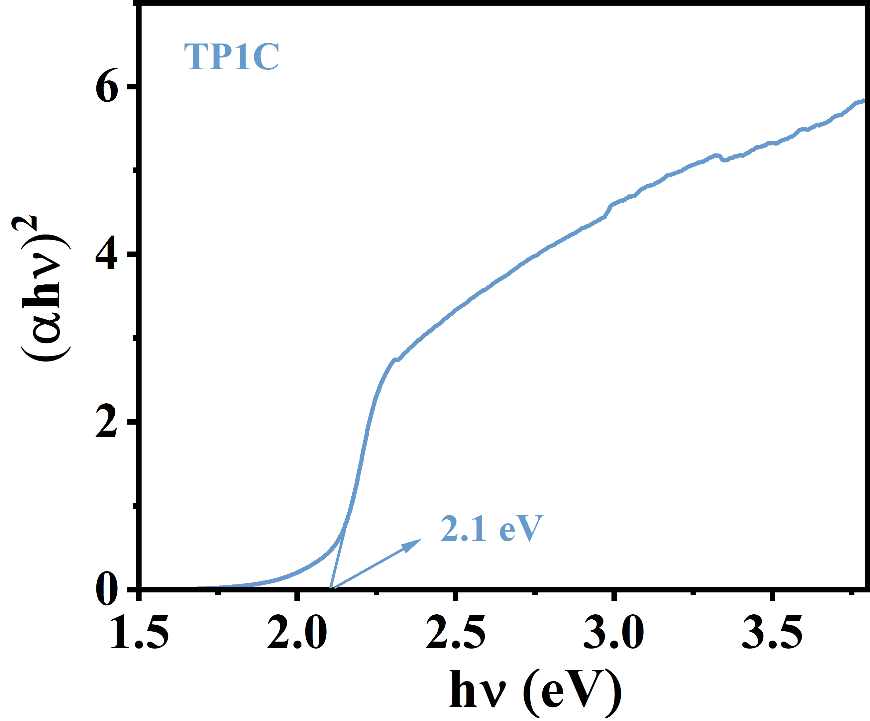


**Fig. S18.** Tauc plots of TP1C.


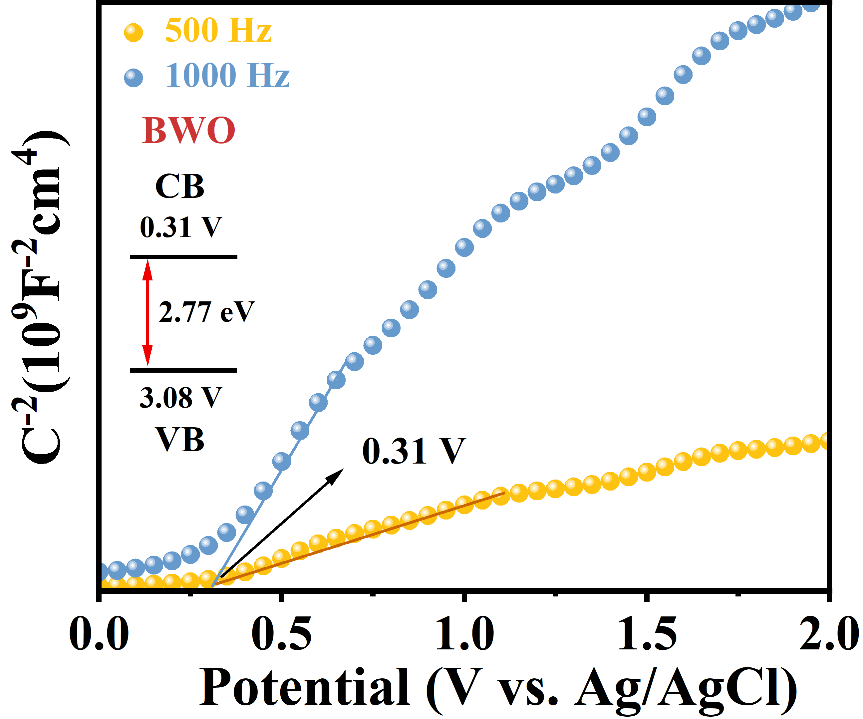


**Fig. S19.** Mott-Schottky plot of BWO.


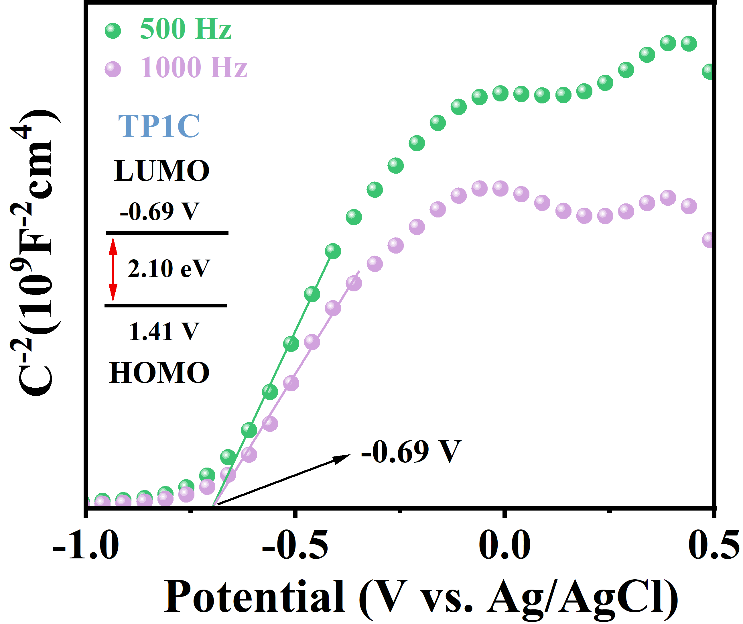


**Fig. S20.** Mott-Schottky plot of TP1C.


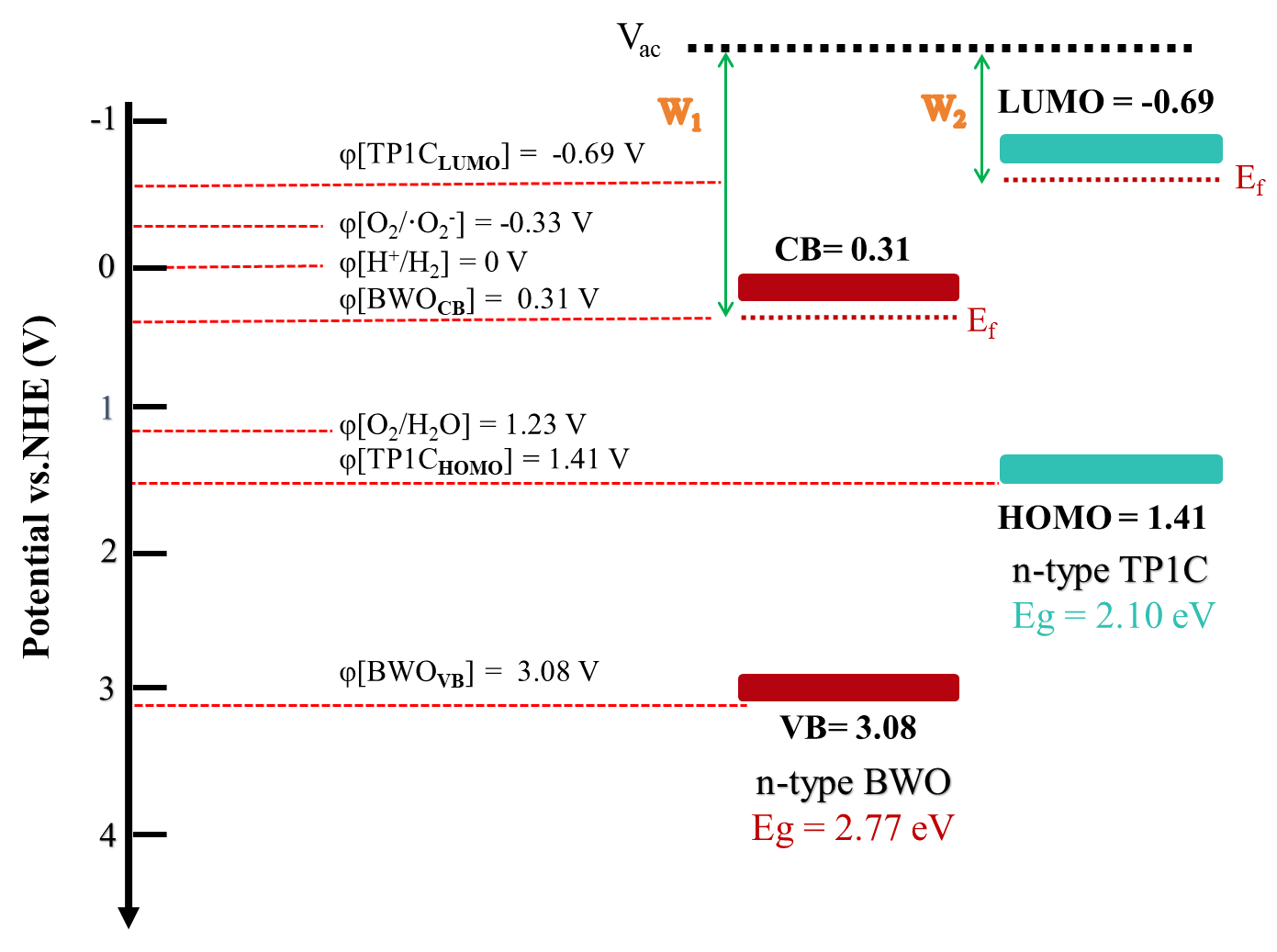


**Fig. S21.** Schematic energy-band image for TP1C and BWO (V_ac_, HOMO, LUMO, CB, VB, E_f_ and W stand for Vacuum level, Highest Occupied Molecular Orbit, Lowest Unoccupied Molecular Orbit, Conduction Band, Valence Band, Fermi level, Work function, respectively).

.

.
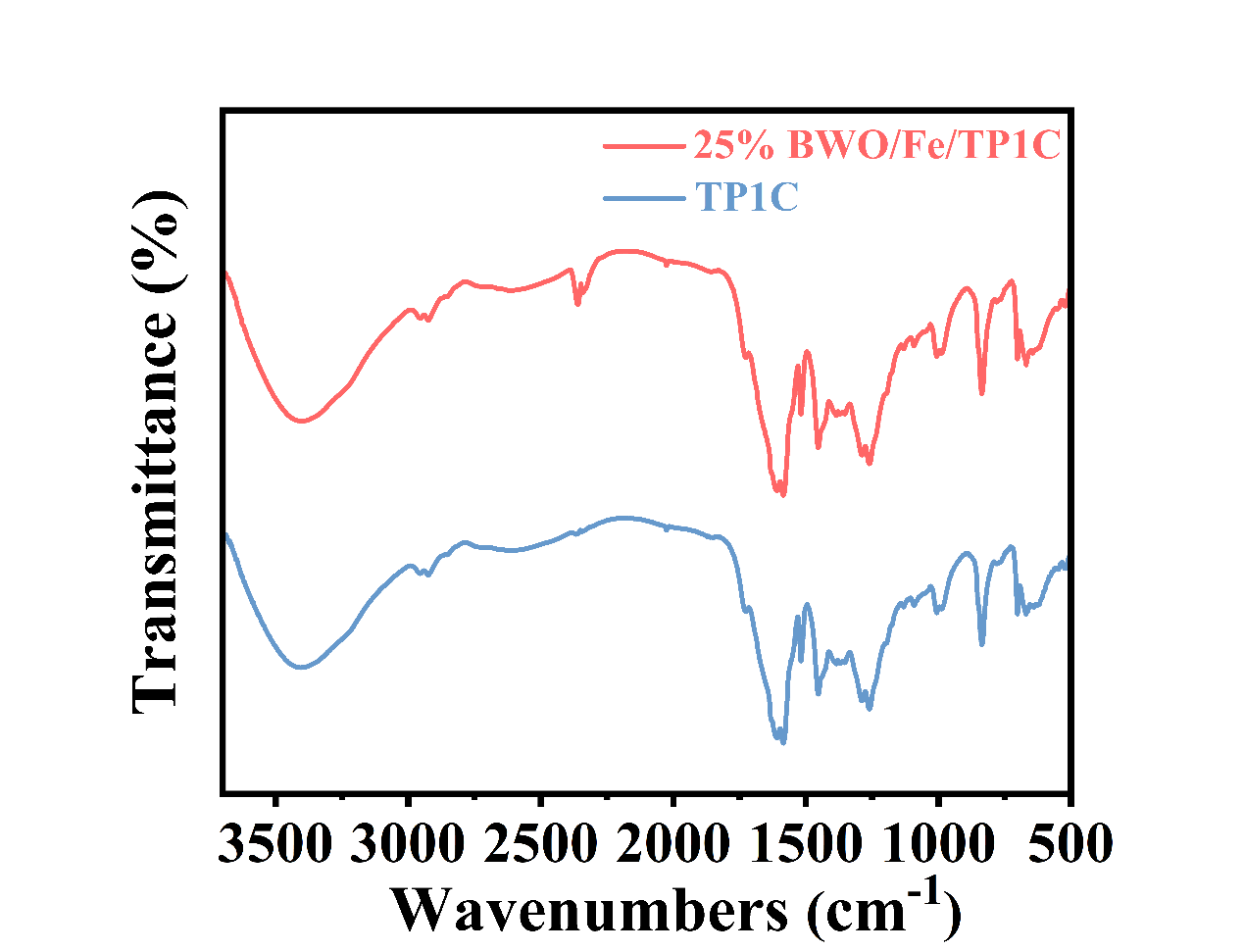


**Fig. S22.** Fourier-transform infrared (FT-IR) spectra of TPC and 25% BWO/Fe/TPC.

As illustrated in Fig. S22, the broad spectral region spanning 3000 to 3700 cm^-1^ corresponds to the stretching vibrations of -OH groups. The distinct peaks located near 1616 cm^-1^ and 1451 cm^-1^ are attributed to the stretching vibrations of C=O and C=C bonds, respectively. Additionally, the band observed around 1255 cm^-1^ is likely associated with the stretching vibrations of C-N bonds.^[11]^


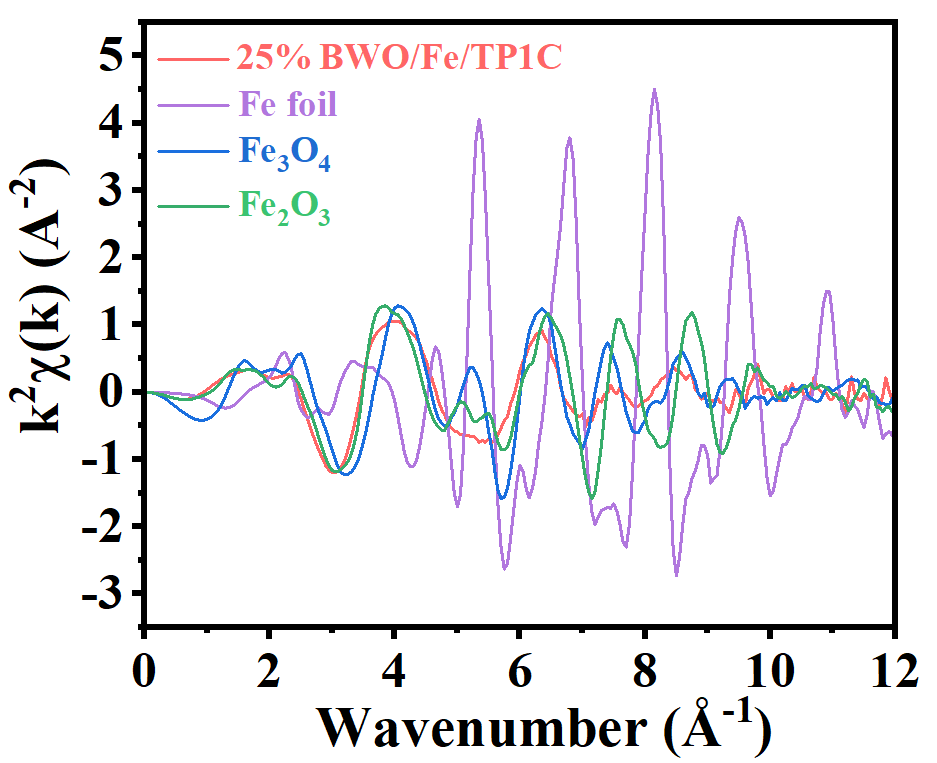


**Fig. S23.** EXAFS spectra for the k space.


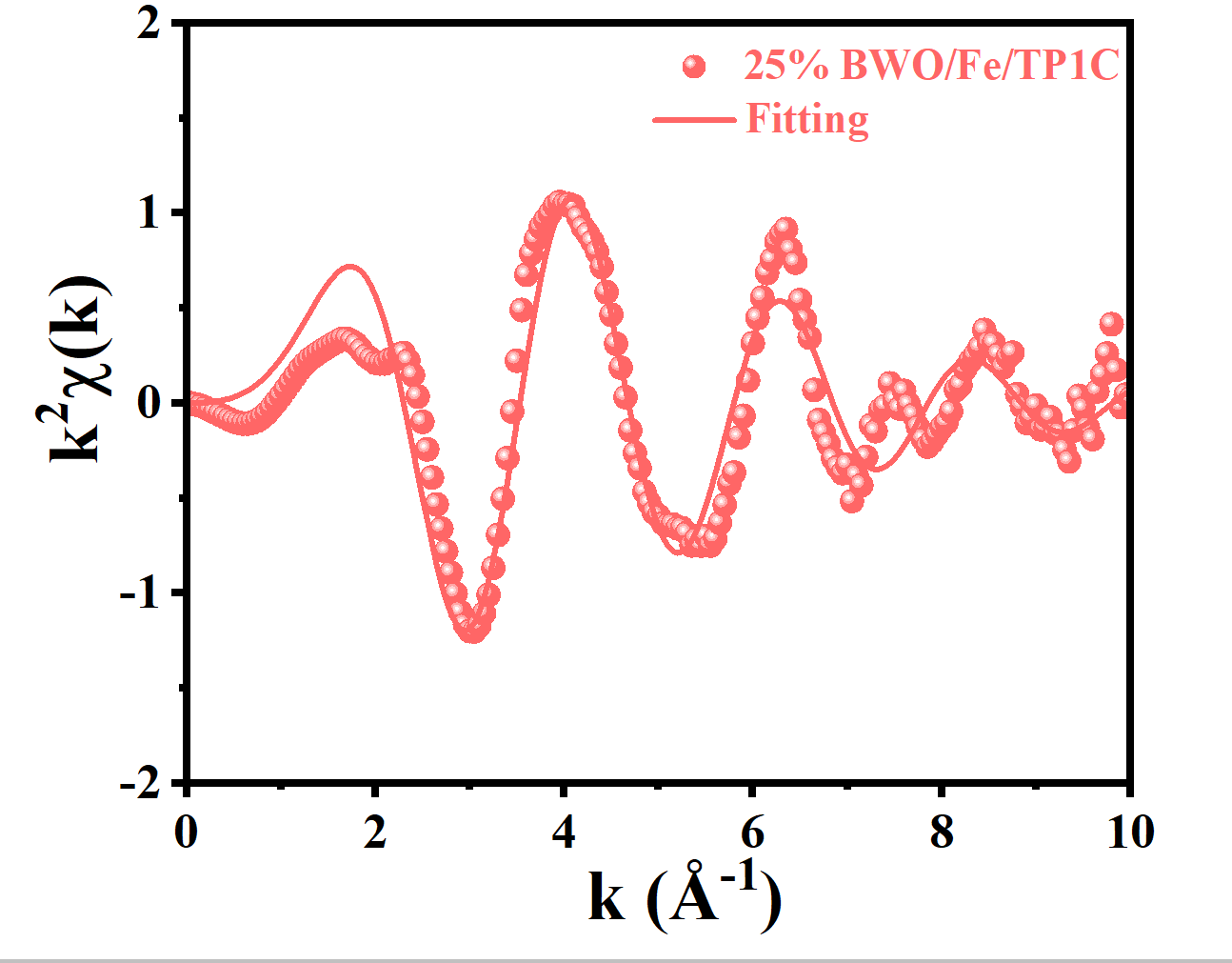


**Fig. S24.** EXAFS fitting spectra for the k space.


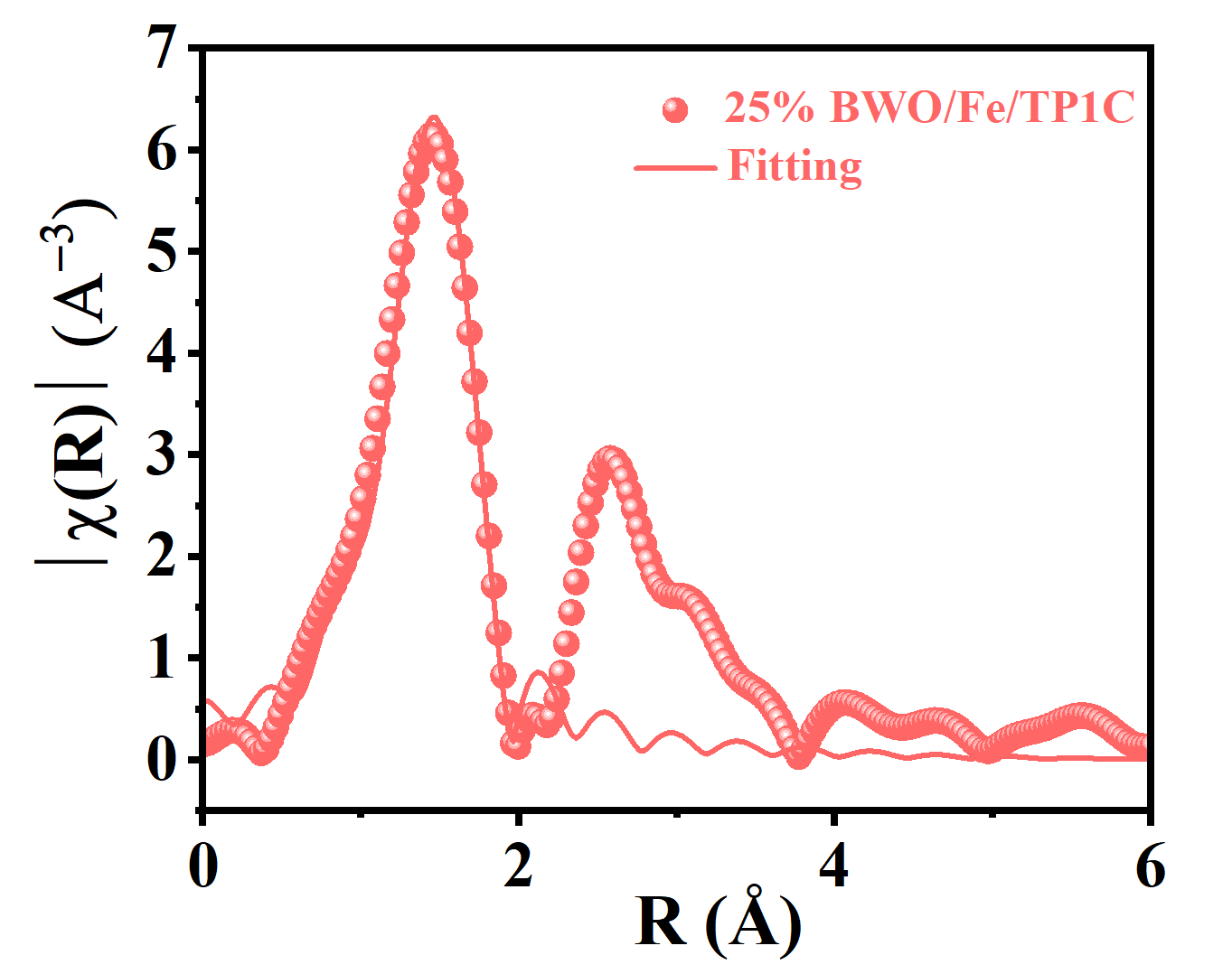


**Fig. S25.** EXAFS fitting spectra for the r space. (EXAFS fitting focused on the first coordination shell to extract key parameters of the absorber atom’s nearest-neighbor environment).


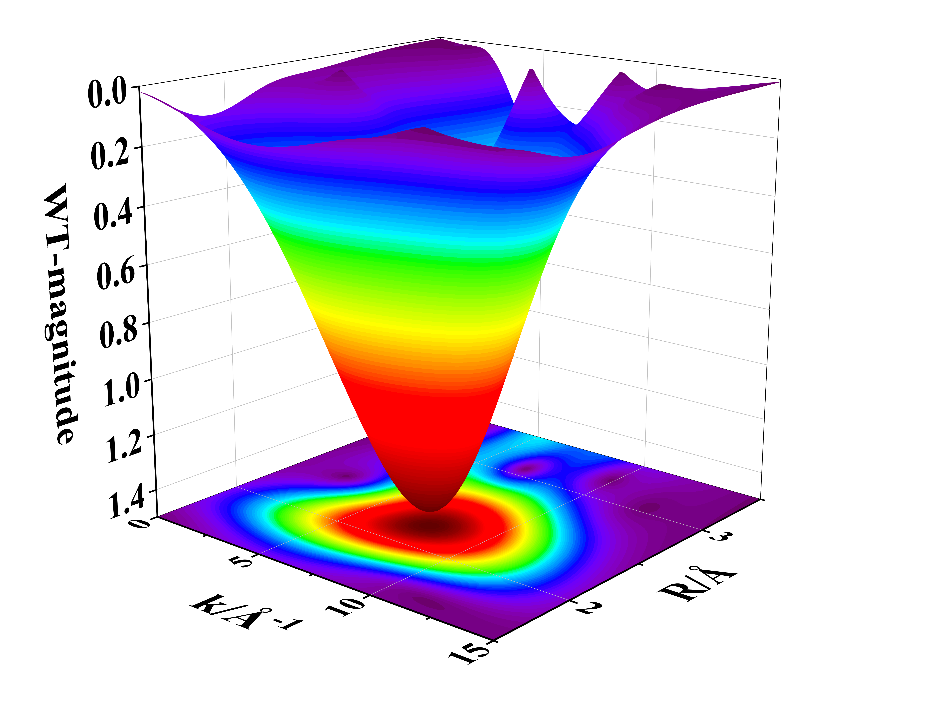


**Fig. S26.** The morlet wavelet transformed X-ray absorption spectra of the Fe foil.


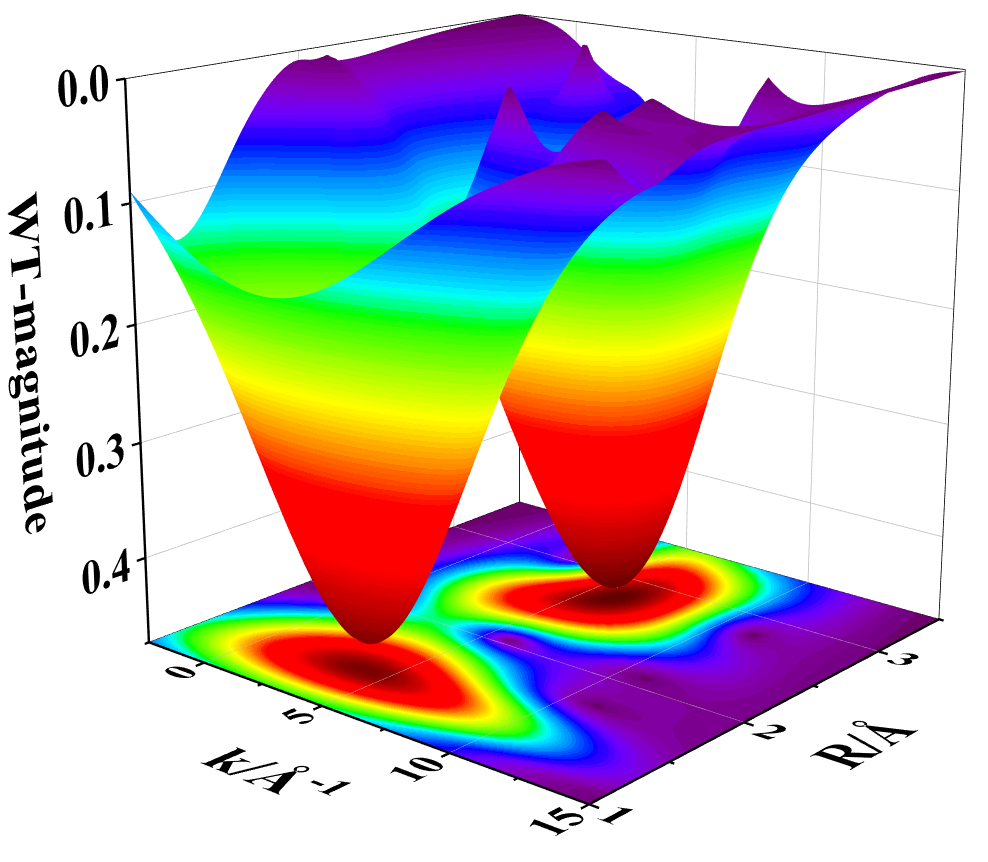


**Fig. S27.** The morlet wavelet transformed X-ray absorption spectra of the Fe_3_O_4_.


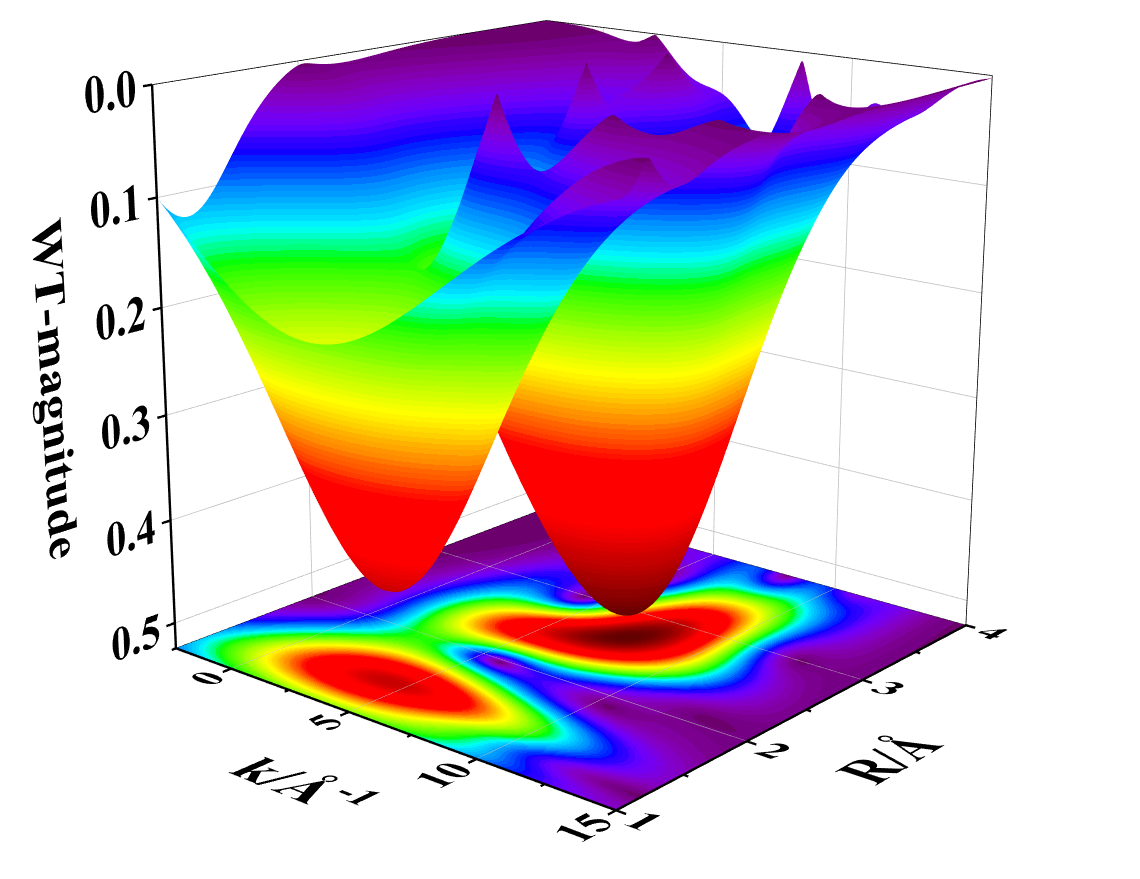


**Fig. S28.** The morlet wavelet transformed X-ray absorption spectra of the Fe_2_O_3_.


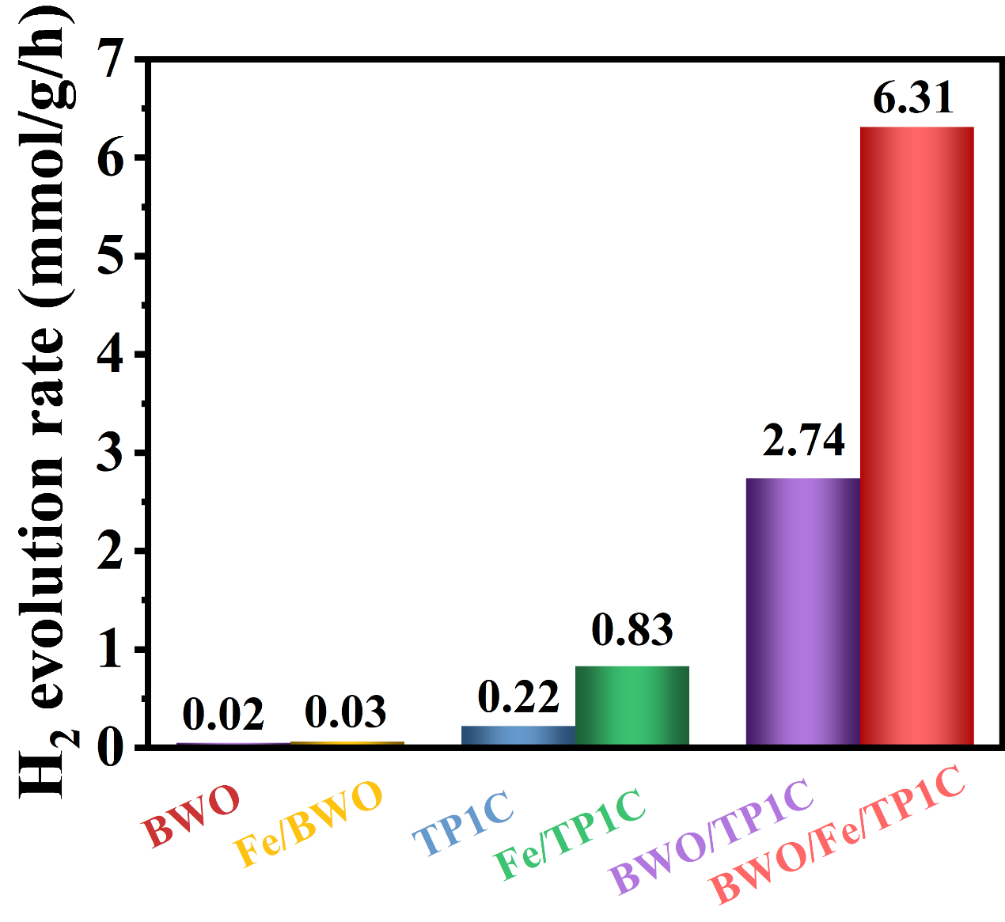


**Fig. S29.** The photocatalytic activity of BWO, Fe/BWO, TP1C, Fe/TP1C, BWO/TP1C and BWO/Fe/TP1C.

As shown in Fig. S29, BWO showed ultra-low photocatalytic activity because its conduction band (CB) potential (0.31 V vs. NHE) is higher (more positive) than the reduction potential of H^+^/H_2_ (0 V vs. NHE), which is thermodynamically incapable of driving the hydrogenolysis reaction.Therefore, the introduction of Fe ions is difficult to improve the photocatalytic activity of BWO. TP1C also displayed relatively low photocatalytic hydrogen production activity due to severe carrier recombination. Fe/TP1C exhibited a 3.8-fold enhancement in hydrogen evolution activity (0.83 mmol/g/h) compared to pure TP1C.


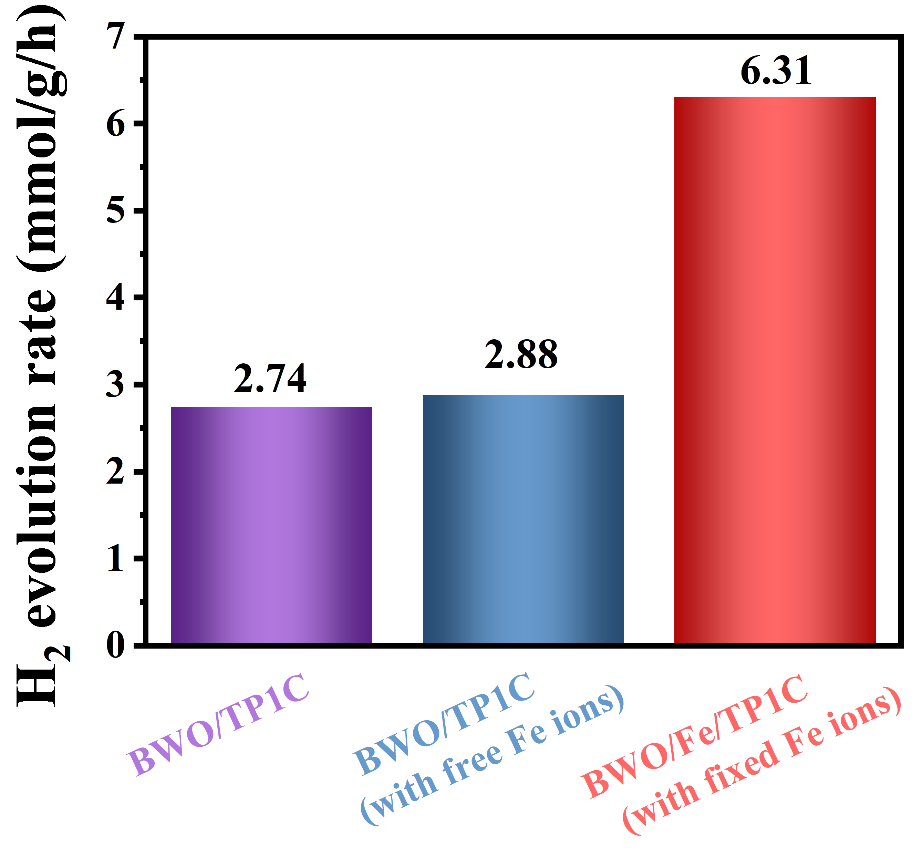


**Fig. S30.** The photocatalytic activity of prepared materials.

The control experiments showed that the free Fe ions in the solution had very limited improvement on the photocatalytic activity of 25% BWO/TP1C. When Fe ions were immobilized on the skeleton of COFs, 25% BWO/Fe/TP1C exhibited significantly enhanced photocatalytic activity, approximately 2.3 times that of 25% BWO/TP1C.


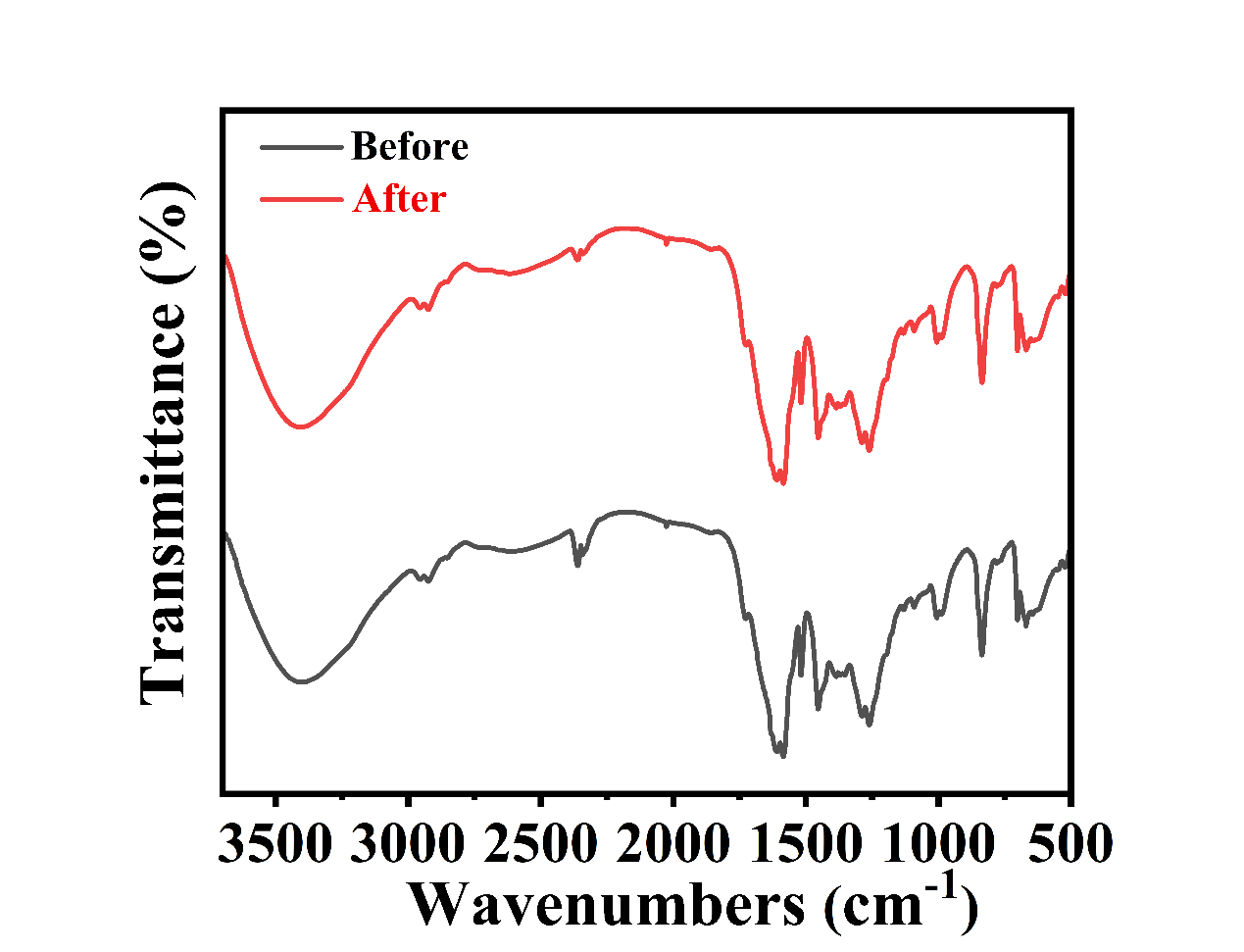


**Fig. S31.** Infrared spectra of 25% BWO/Fe/TP1C before and after the photocatalytic reaction.

As shown in Fig. S31, FT-IR analysis was conducted on 25% BWO/Fe/TP1C after the photocatalytic reaction, and the peak positions remained nearly unchanged compared to those before the reaction, confirming its excellent stability.


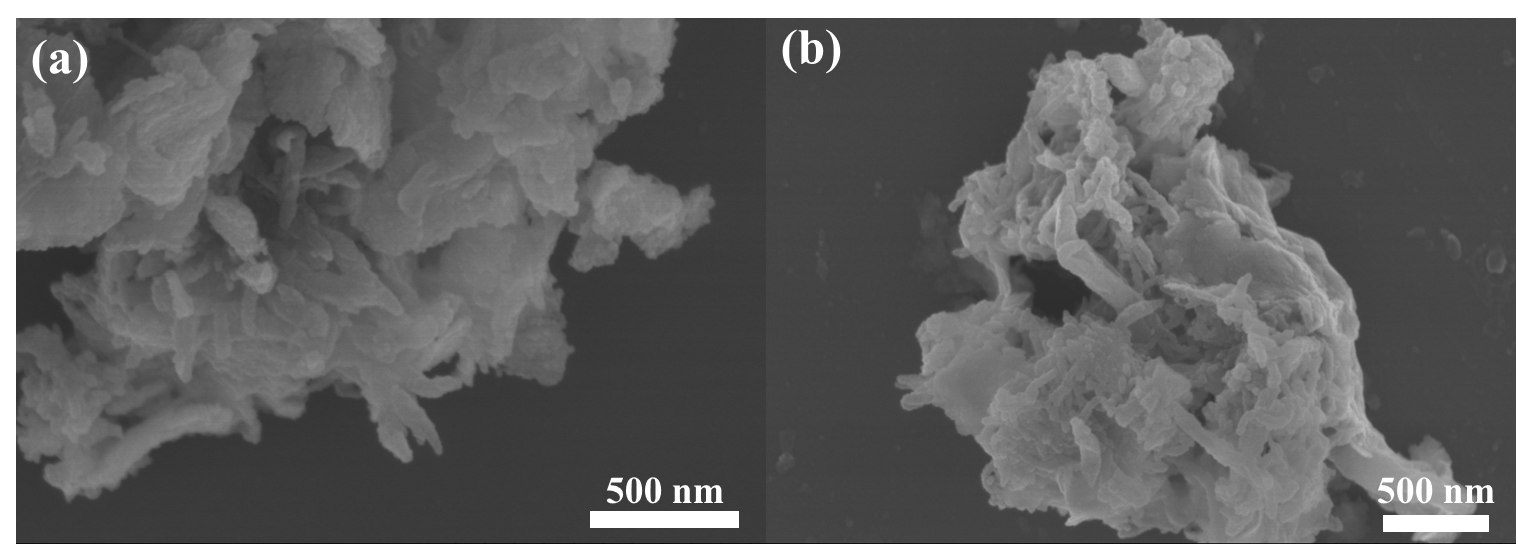


**Fig. S32.** (a) The SEM image of BWO/Fe/TP1C before photocatalytic reaction. (b) The SEM image of BWO/Fe/TP1C after photocatalytic reaction.

As shown in Fig. S32, the morphology and structure of BWO/Fe/TP1C hardly changed after the photocatalytic reaction, indicating its good catalytic stability.


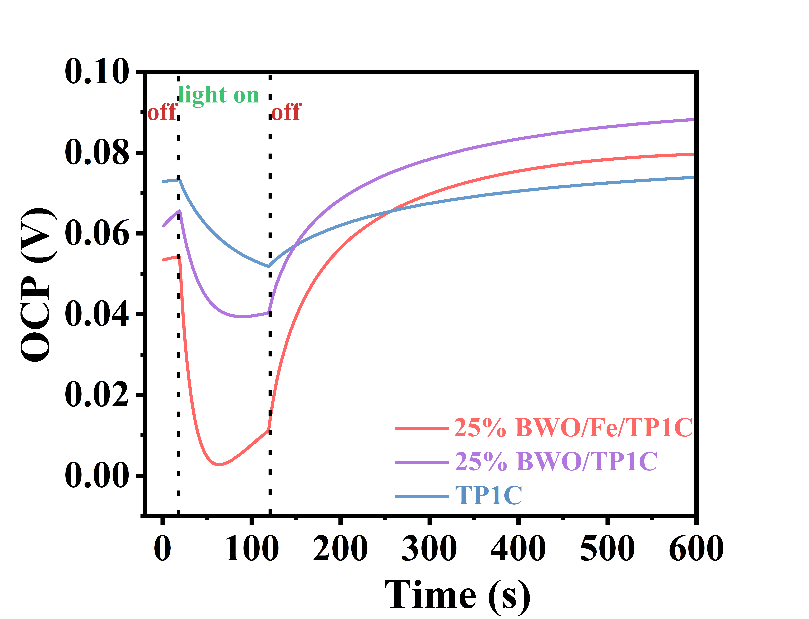


**Fig. S33.** The OCVD experiments for TP1C, 25% BWO/TP1C and 25% BWO/Fe/TP1C.


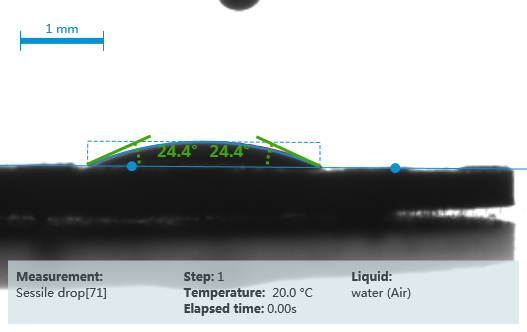


**Fig. S34**. The water contact angle of TP1C.

As displayed in Fig. S34, the water contact angle measurements demonstrated a certain degree of hydrophilicity of pure TP1C.

**
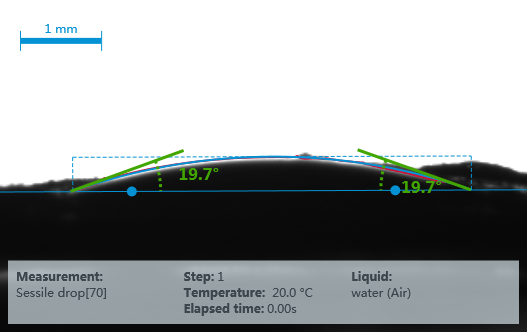
**

**Fig. S35**. The water contact angle of 25% BWO/TP1C.

As shown in Fig. S35, compared with bare TP1C, BWO/TP1C exhibited relatively more hydrophilicity.


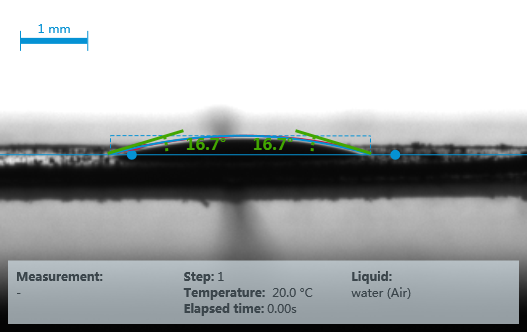


**Fig. S36.** The water contact angle of 25% BWO/Fe/TP1C.

As depiced in Fig. S36, compared with bare TP1C and 25% BWO/TP1C, 25% BWO/Fe/TP1C owned the smaller water contact angle, proving its stronger hydrophilicity.


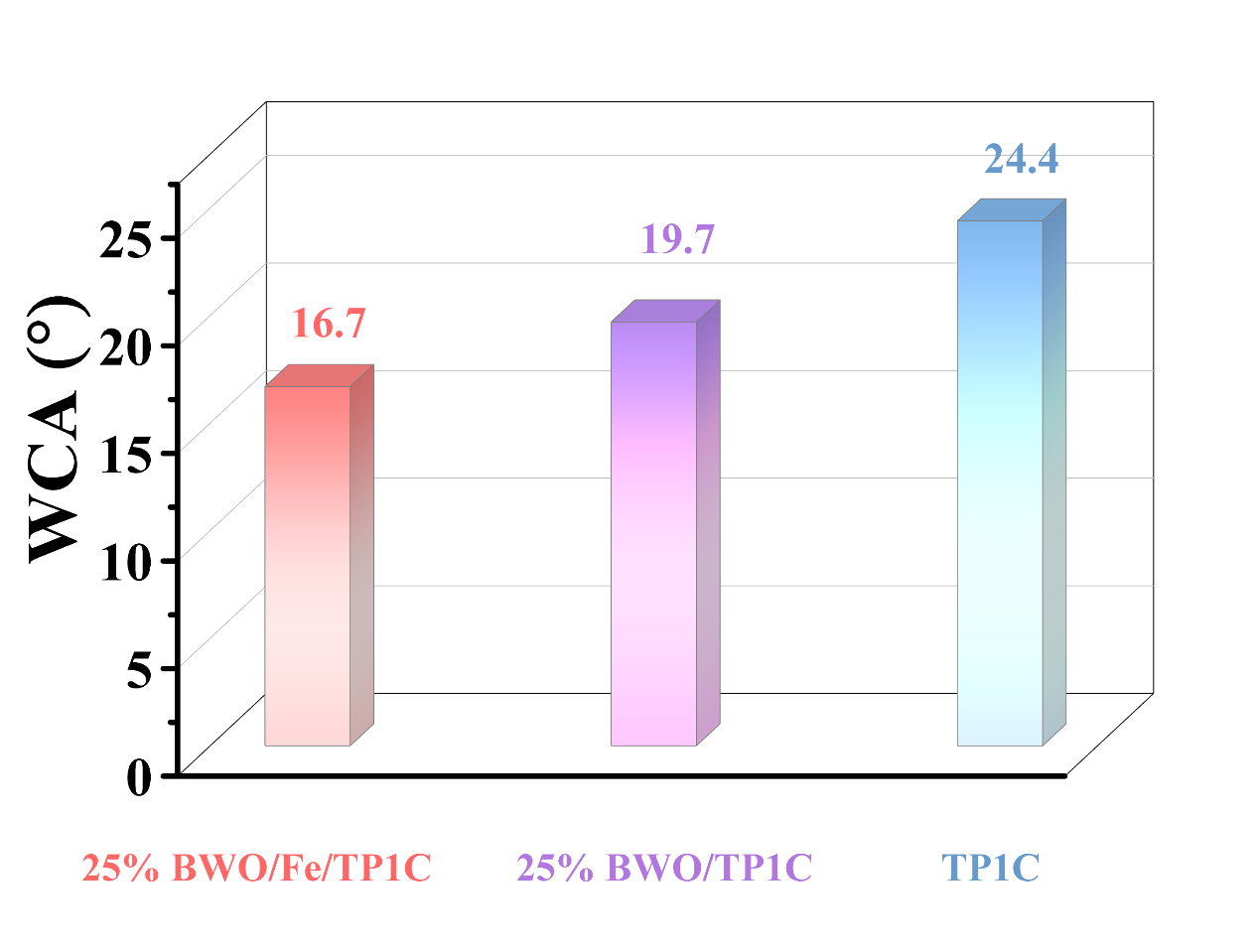


**Fig. S37.** The water contact angles of TP1C, 25% BWO/TP1C and 25% BWO/Fe/TP1C.

The 25% BWO/Fe/TP1C hybrid sample, exhibiting a smaller water contact angle than that of bare TP1C and 25% BWO/TP1C, clearly showed significantly greater hydrophilicity, suggesting its capability for better substrate enrichment and improved promotion of photocatalytic activity for H_2_ generation.


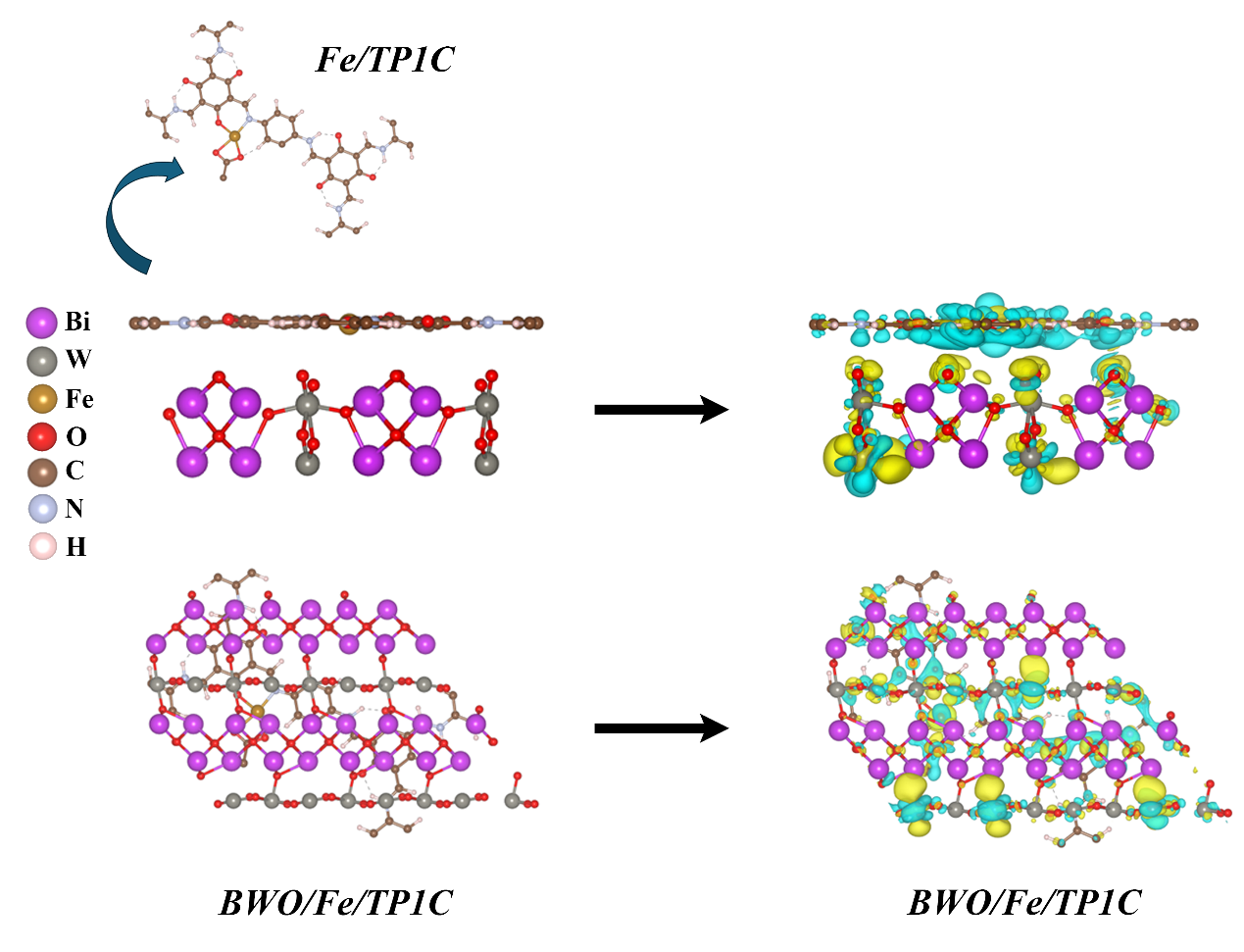


**Fig. S38.** The calculated charge density difference within BWO/Fe/TP1C.


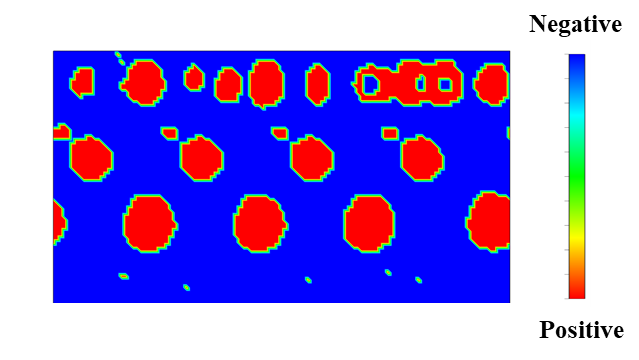


**Fig. S39.** The longitudinal section depiction of 25% BWO/Fe/TP1C in figure 5f.


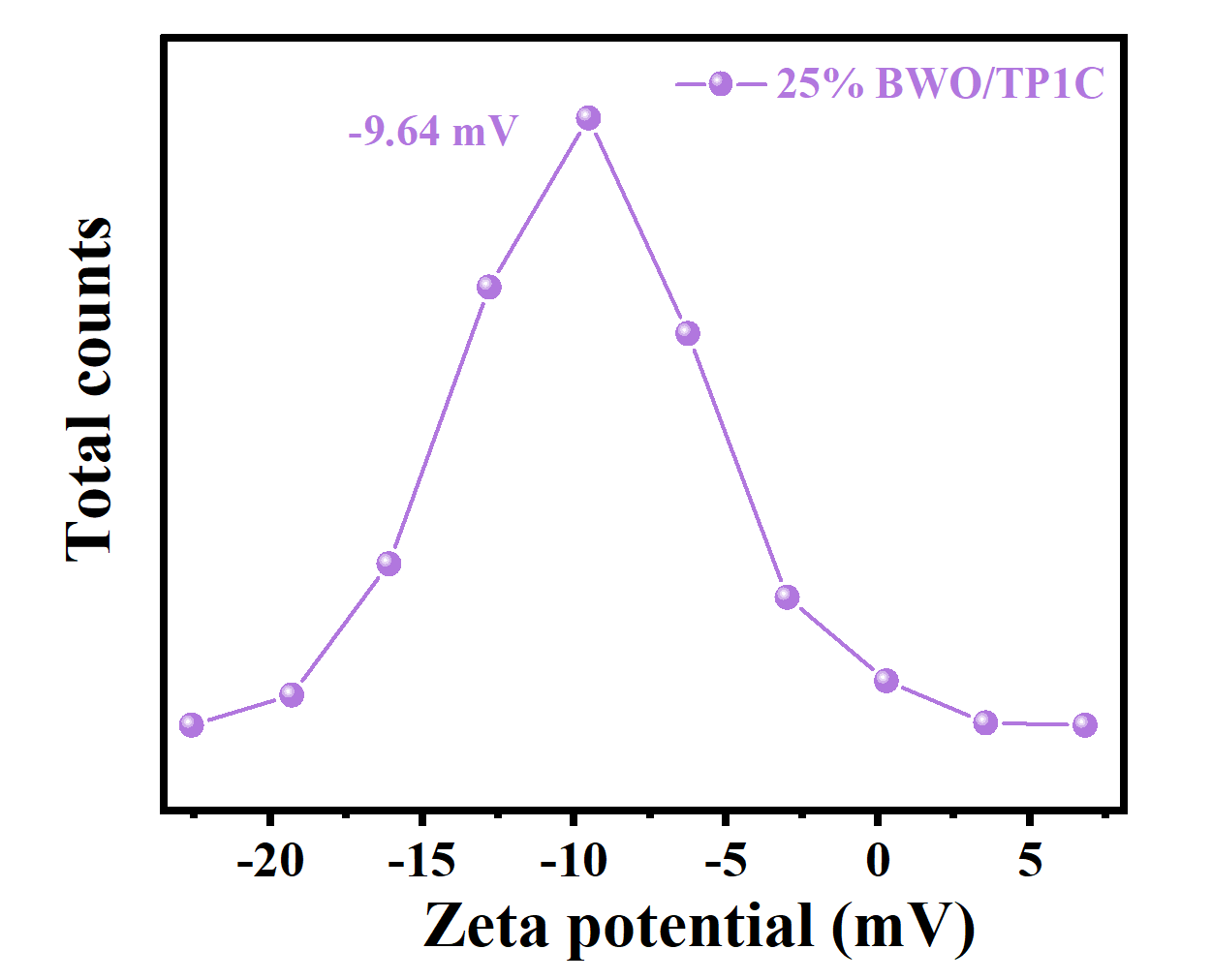


**Fig. S40.** The Zeta potential of 25% BWO/TP1C.

As shown in Fig. S40, the Zeta potential of 25% BWO/TP1C is about -9.64 mV.


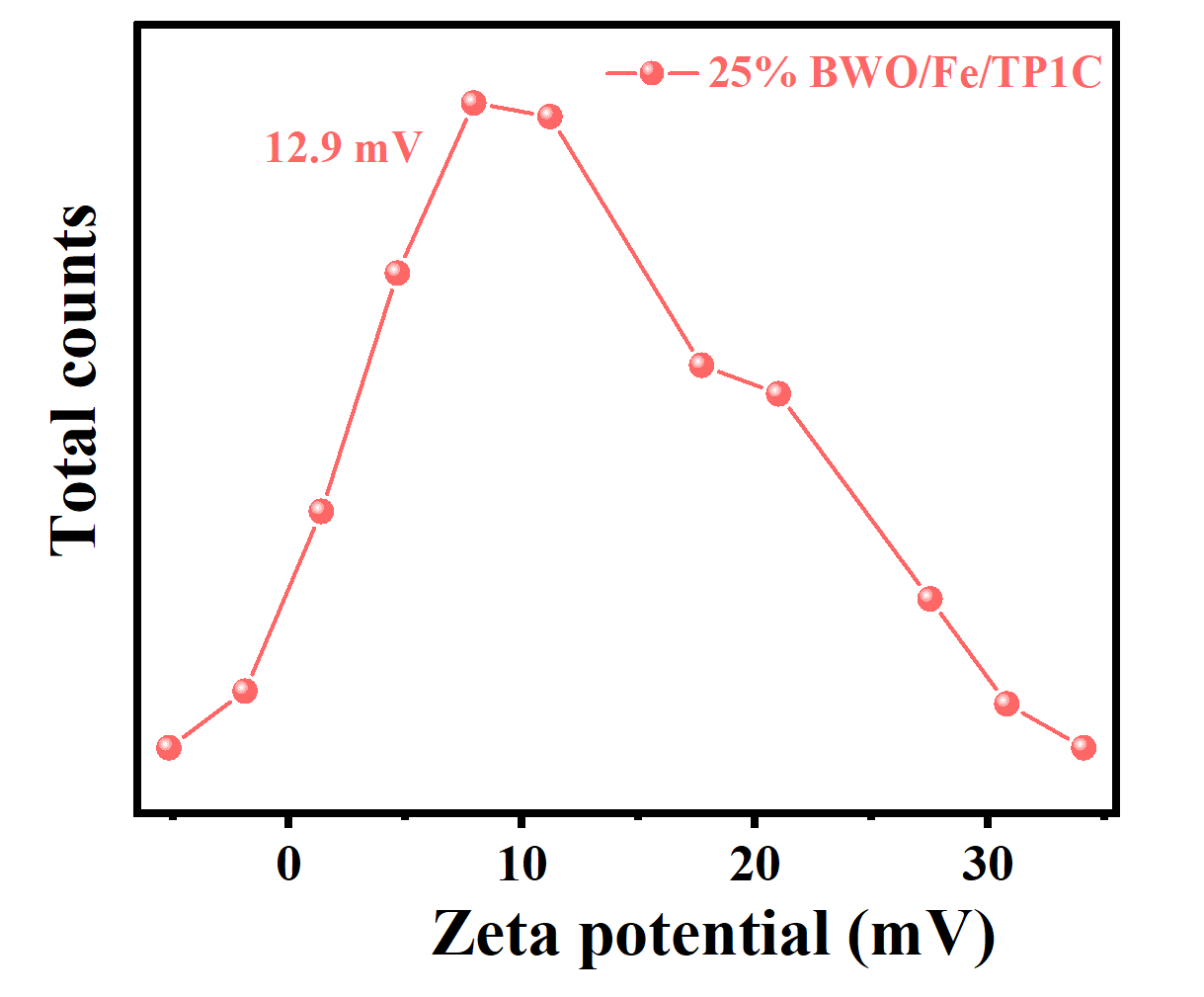


**Fig. S41.** The Zeta potential of 25% BWO/Fe/TP1C.

As shown in Fig. S41, the Zeta potential of 25% BWO/Fe/TP1C is about 12.9 mV.


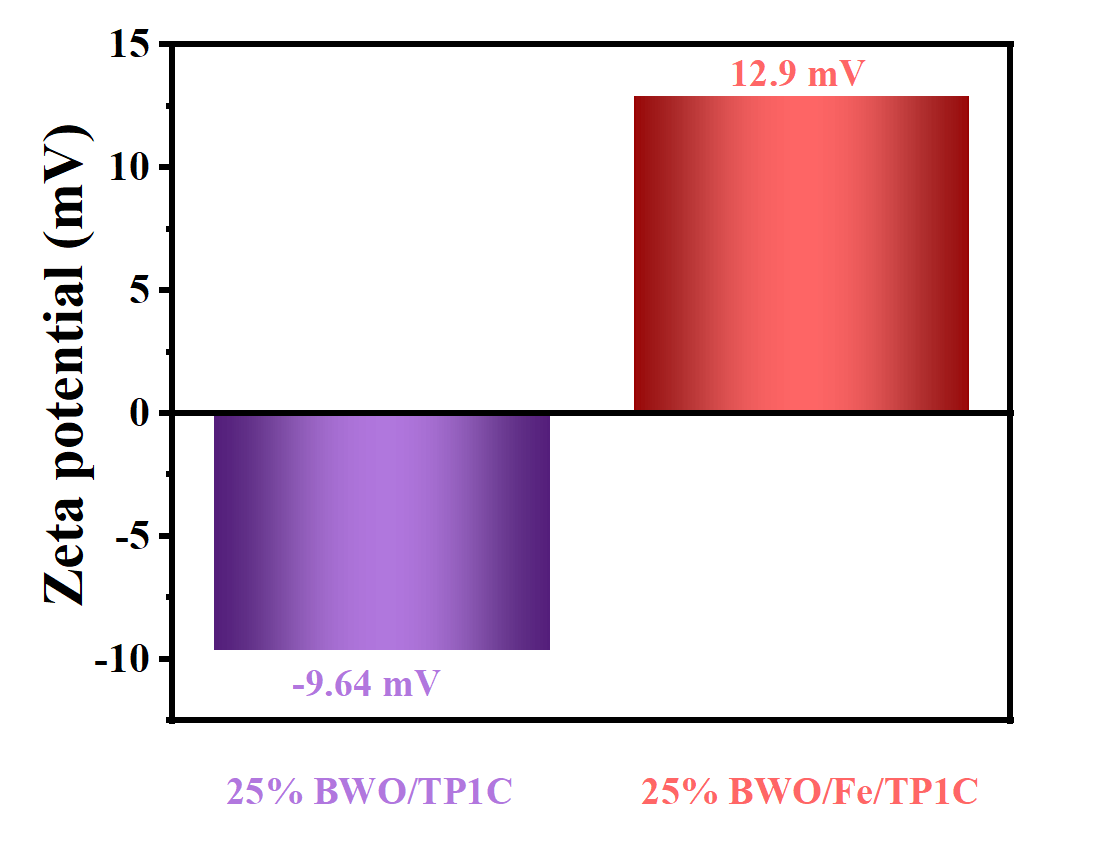


**Fig. S42.** The Zeta potential of 25% BWO/TP1C and 25% BWO/Fe/TP1C.

As shown in Fig. S42, compared with 25% BWO/TP1C, 25% BWO/Fe/TP1C exhibits a larger Zeta potential, indicating the formation of a stronger internal electric field.


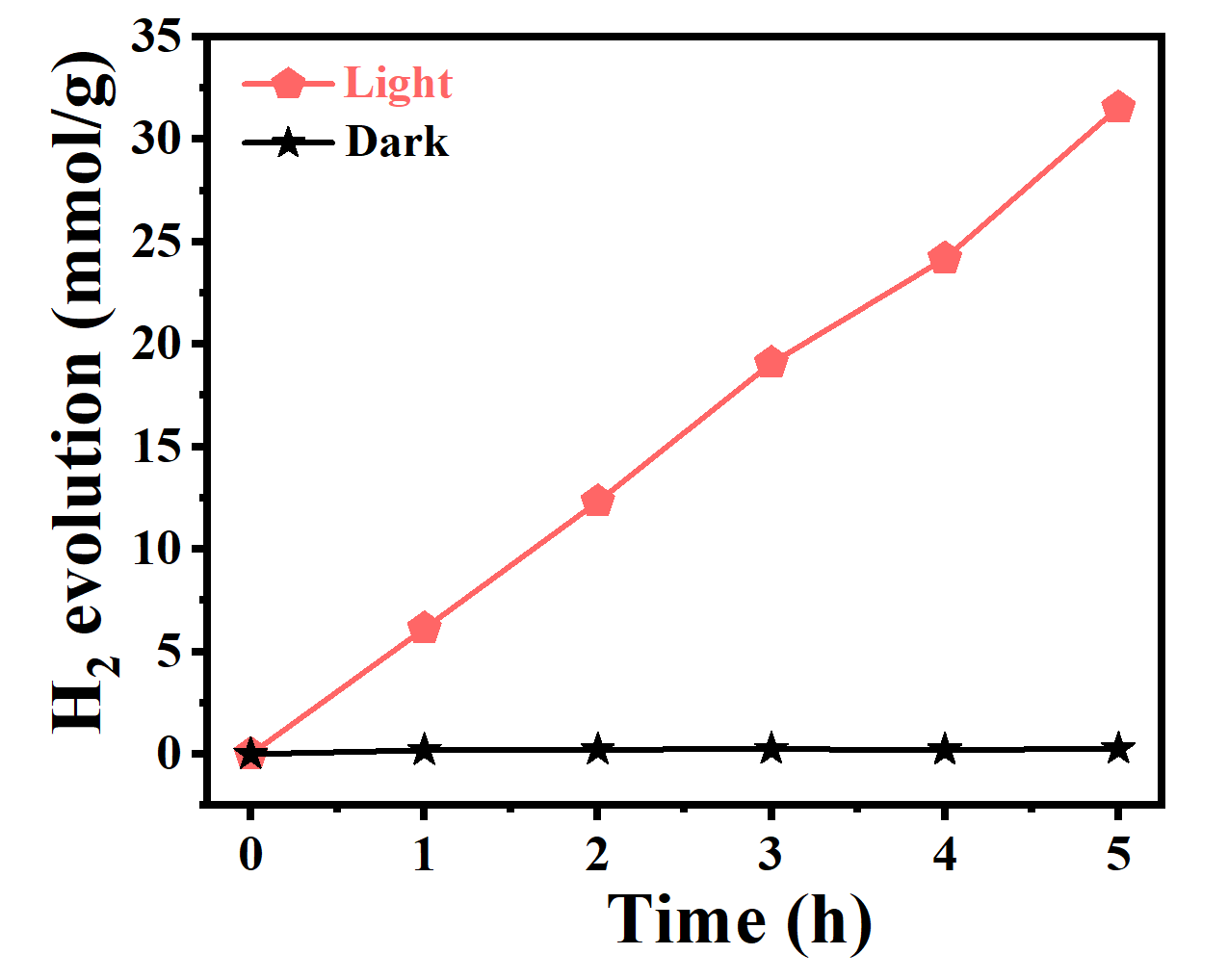


**Fig. S43.** The photocatalytic activity of 25% BWO/Fe/TP1C under light or dark conditions.

As shown in Fig. S43, 25% BWO/Fe/TP1C displayed almost no photocatalytic activity under dark conditions.


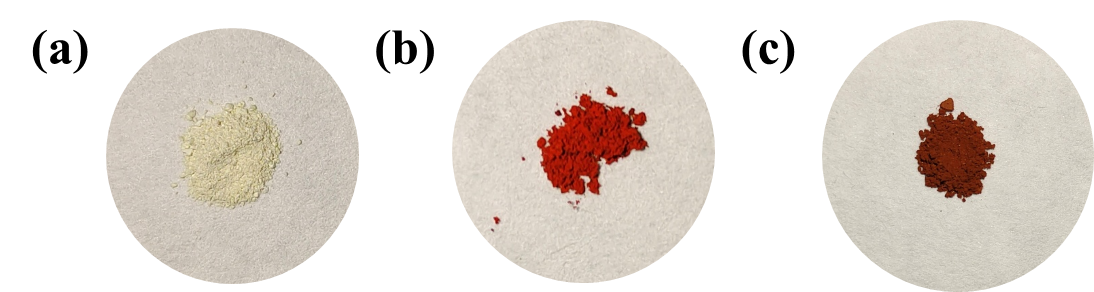


**Fig. S44.** The prepared powder samples. (a) BWO, (b) TP1C, (c) 25% BWO/Fe/TP1C.

**Tab. S1.** The weight ratio of each element in Fe/BWO

| Element | O | Bi | W | Fe | Totals |
| --- | --- | --- | --- | --- | --- |
| Weight% | 6.91 | 20.68 | 72.28 | 0.13 | 100.00 |

**Tab. S2.** The weight ratio of each element in Fe/TP1C

| Element | C | N | O | Fe | Totals |
| --- | --- | --- | --- | --- | --- |
| Weight% | 44.47 | 29.17 | 20.89 | 5.47 | 100.00 |

**Tab. S3.** The BET surface area of prepared samples

| Sample | BET surface area (m^2^**·**g^-1^) |
| --- | --- |
| TP1C | 1035.8 |
| 25% BWO/Fe/TP1C | 750.9 |

**Tab. S4.** Fe K-edge EXAFS curves Fitting Parameters.

| Samples | Path | CN^[a]^ | R_eff_ (Å)^[b]^ | ΔE_0_(eV)^[c]^ | σ^2[d]^ | Δr(Å)^[e]^ | R^[f]^ |
| --- | --- | --- | --- | --- | --- | --- | --- |
| 25% BWO/Fe/TP1C | Fe-O | 4.79 | 1.97 | -5.08 | 0.00965 | 0.0073 | 1.6% |

[a] Coordination number, [b] Interatomic distance, [c] Edge energy shift, [d] Debye-Waller factor, [e] Interatomic distance error, [f] R factor.

**Tab. S5.** The lifetime of prepared samples in time-resolved PL test.

| Sample | τ_1_ (ns) | B_1_ | τ_2_ (ns) | B_2_ | τ_3_ (ns) | B_3_ | τ_av_ (ns) |
| --- | --- | --- | --- | --- | --- | --- | --- |
| TP1C | 0.35 | 16177.80 | 3.58 | 178.08 | 19.76 | 6.29 | 1.04 |
| 25% BWO/TPC | 0.48 | 11124.41 | 1.83 | 998.57 | 18.37 | 9.26 | 1.23 |
| 25% BWO/Fe/TPC | 0.97 | 8750.30 | 4.0 | 237.95 | 13.27 | 58.56 | 2.19 |

**Tab. S6.** Photocatalytic activity of reported photocatalysts and this work.

| **Sample** | **Light** | **Sacrificial agent** | **Cocatalyst** | **H_2_ evolution rate**  **(mmol·g^-1^·h^-1^)** | **Ref** |
| --- | --- | --- | --- | --- | --- |
| 25% BWO/Fe/TP1C | λ ≥ 420 nm | Ascorbic acid | - | 6.31 | This work |
| CdS-COF | λ ≥ 420 nm | Lactic acid | Pt | 3.68 | [12] |
| CYANO-CON | λ ≥ 420 nm | Ascorbic acid | - | 0.02 | [13] |
| PhBp-CTF-Ir | λ ≥ 420 nm | TEOA | - | 0.24 | [14] |
| Py-ClTP-BT-COF | λ ≥ 420 nm | Ascorbic acid | - | 2.20 | [15] |
| TPCBP B-COF | λ ≥ 420 nm | TEOA | - | 0.08 | [16] |
| Fe_2_O_3_/TpPa-2-COF | λ ≥ 420 nm | Sodium ascorbate | - | 3.77 | [17] |
| BTTh-TZ-COF | λ ≥ 420 nm | Ascorbic acid | Pt | 5.22 | [18] |
| COF-OH-2 | λ ≥ 420 nm | Ascorbic acid | Pt | 2.91 | [19] |
| MoS_2_-TpPa-1-COF | λ ≥ 420 nm | Ascorbic acid | - | 5.59 | [20] |
| TAPFy-PhI COF | λ ≥ 420 nm | Ascorbic acid | Pt | 2.72 | [21] |
| ODA-COF | λ ≥ 420 nm | Ascorbic acid | Pt | 2.62 | [22] |
| Ni-Bn-COF | λ ≥ 420 nm | Ascorbic acid | Pt | 2.80 | [23] |
| COF/ZIS | λ ≥ 420 nm | Na_2_S/Na_2_SO_3_ | **-** | 0.85 | [24] |
| BT-TAPT COF | λ ≥ 420 nm | Ascorbic acid | **-** | 0.01 | [25] |
| TP-BDDA-COF | λ ≥ 420 nm | TEOA | Pt | 1.81 | [26] |
| Tz-COF-4 | λ ≥ 420 nm | Ascorbic acid | Pt | 4.30 | [27] |
| BTH-2 | λ ≥ 420 nm | Ascorbic acid | Pt | 1.20 | [28] |
| N_3_-COF | λ ≥ 420 nm | Sodium ascorbate | Pt | 1.70 | [29] |
| sp^2^-COF_ERDN_ | λ ≥ 420 nm | TEOA | Pt | 2.12 | [30] |
| TTV-COF | λ ≥ 420 nm | Ascorbic acid | Pt | 5.50 | [31] |
| TpPa-COF-(CH_3_)_2_ | λ ≥ 420 nm | Sodium ascorbate | - | 0.07 | [32] |

**References**

[1] Y. He, D. Wang, X. Li, Q. Fu, L. Yin, Q. Yang and H. Chen, *Chemosphere* **2021**, *284*, 131386.

[2] J. Zhang, X. Li, H. Hu, H. Huang, H. Li, X. Sun and T. Ma, *Nat. Commun.* **2024**, *15*, 9576.

[3] Y. G. Lei, K. H. Ng, Y. C. Zhu, Y. Z. Zhang, Z. X. Li, S. Xu, J. Y. Huang, J. Hu, Z. Chen, W. L. Cai and Y. K. Lai, *Chem. Eng. J.* **2023**, *452*, 139325.

[4] Z. P. Qian, R. Zhang, H. J. Hu, Y. Xiao, H. Li, X. D. Sun and T. Y. Ma, *Sol. RRL* **2023**, *7*, 2300547.

[5] C. X. Zhang, C. F. Xie, Y. Y. Gao, X. P. Tao, C. M. Ding, F. T. Fan and H. L. Jiang, *Angew. Chem. Int. Ed.* **2022**, *61*, e202204108.

[6] P. Giannozzi, O. Andreussi, T. Brumme, O. Bunau, M. Buongiorno Nardelli, M. Calandra, R. Car, C. Cavazzoni, D. Ceresoli, M. Cococcioni, N. Colonna, I. Carnimeo, A. Dal Corso, S. de Gironcoli, P. Delugas, R. A. DiStasio, A. Ferretti, A. Floris, G. Fratesi, G. Fugallo, R. Gebauer, U. Gerstmann, F. Giustino, T. Gorni, J. Jia, M. Kawamura, H. Y. Ko, A. Kokalj, E. Küçükbenli, M. Lazzeri, M. Marsili, N. Marzari, F. Mauri, N. L. Nguyen, H. V. Nguyen, A. Otero-de-la-Roza, L. Paulatto, S. Poncé, D. Rocca, R. Sabatini, B. Santra, M. Schlipf, A. P. Seitsonen, A. Smogunov, I. Timrov, T. Thonhauser, P. Umari, N. Vast, X. Wu and S. Baroni, *J. Phys.: Condens. Matter* **2017**, *29*, 465901.

[7] P. Giannozzi, S. Baroni, N. Bonini, M. Calandra, R. Car, C. Cavazzoni, D. Ceresoli, G. L. Chiarotti, M. Cococcioni, I. Dabo, A. Dal Corso, S. de Gironcoli, S. Fabris, G. Fratesi, R. Gebauer, U. Gerstmann, C. Gougoussis, A. Kokalj, M. Lazzeri, L. Martin-Samos, N. Marzari, F. Mauri, R. Mazzarello, S. Paolini, A. Pasquarello, L. Paulatto, C. Sbraccia, S. Scandolo, G. Sclauzero, A. P. Seitsonen, A. Smogunov, P. Umari and R. M. Wentzcovitch, *J. Phys.: Condens. Matter* **2009**, *21*, 395502.

[8] J. Paier, R. Hirschl, M. Marsman and G. Kresse, *J. Chem. Phys.* **2005**, *122*, 234102.

[9] S. Grimme, S. Ehrlich and L. Goerigk, *J. Comput. Chem.* **2011**, *32*, 1456-1465.

[10] S. Grimme, J. Antony, S. Ehrlich and H. Krieg, *J. Chem. Phys.* **2010**, *132*, 154104.

[11] W. J. Weng and J. Guo, *Nat. Commun.* **2022**, *13*, 5768.

[12] J. Thote, H. B. Aiyappa, A. Deshpande, D. Díaz Díaz, S. Kurungot and R. Banerjee, *Chem. - Eur. J.* **2014**, *20*, 15961-15965.

[13] C. Li, J. Liu, H. Li, K. Wu, J. Wang and Q. Yang, *Nat. Commun.* **2022**, *13*, 2357.

[14] N. Xu, Y. Diao, Z. Xu, H. Ke and X. Zhu, *ACS Appl. Energy Mater.* **2022**, *5*, 7473-7478.

[15] W. Chen, L. Wang, D. Mo, F. He, Z. Wen, X. Wu, H. Xu and L. Chen, *Angew. Chem. Int. Ed.* **2020**, *59*, 16902-16909.

[16] S. Altınışık, G. Yanalak, İ. Hatay Patır and S. Koyuncu, *ACS Appl. Mater. Interfaces* **2023**, *15*, 18836-18844.

[17] W. Li, Z. Gong, X. Yan, D. Wang, J. Liu, X. Guo, Z. Zhang and G. Li, *J. Mater. Chem. A* **2020**, *8*, 433-442.

[18] H. Liu, X. Zheng, J. Xu, X. Jia, M. Chao, D. Wang and Y. Zhao, *ACS Appl. Mater. Interfaces* **2023**, *15*, 16794-16800.

[19] Y. Chen, X. Luo, J. Zhang, L. Hu, T. Xu, W. Li, L. Chen, M. Shen, S.-B. Ren, D.-M. Han, G.-H. Ning and D. Li, *J. Mater. Chem. A* **2022**, *10*, 24620-24627.

[20] M.-Y. Gao, C.-C. Li, H.-L. Tang, X.-J. Sun, H. Dong and F.-M. Zhang, *J. Mater. Chem. A* **2019**, *7*, 20193-20200.

[21] G. Zhang, M. Zhao, L. Su, H. Yu, C. Wang, D. Sun and Y. Ding, *ACS Appl. Mater. Interfaces* **2023**, *15*, 20310-20316.

[22] S. Yang, H. Lv, H. Zhong, D. Yuan, X. Wang and R. Wang, *Angew. Chem. Int. Ed.* **2022**, *61*, e202115655.

[23] L. Sun, M. Lu, Z. Yang, Z. Yu, X. Su, Y.-Q. Lan and L. Chen, *Angew. Chem. Int. Ed.* **2022**, *61*, e202204326.

[24] P. Dong, T. Cheng, J.-l. Zhang, J. Jiang, L. Zhang, X. Xi and J. Zhang, *ACS Appl. Energy Mater.* **2023**, *6*, 1103-1115.

[25] G.-B. Wang, S. Li, C.-X. Yan, Q.-Q. Lin, F.-C. Zhu, Y. Geng and Y.-B. Dong, *Chem. Commun.* **2020**, *56*, 12612-12615.

[26] P. Pachfule, A. Acharjya, J. Roeser, T. Langenhahn, M. Schwarze, R. Schomäcker, A. Thomas and J. Schmidt, *J. Am. Chem. Soc.* **2018**, *140*, 1423-1427.

[27] K. Wang, Z. Jia, Y. Bai, X. Wang, S. E. Hodgkiss, L. Chen, S. Y. Chong, X. Wang, H. Yang, Y. Xu, F. Feng, J. W. Ward and A. I. Cooper, *J. Am. Chem. Soc.* **2020**, *142*, 11131-11138.

[28] Y. Wang, W. Hao, H. Liu, R. Chen, Q. Pan, Z. Li and Y. Zhao, *Nat. Commun.* **2022**, *13*, 100.

[29] V. S. Vyas, F. Haase, L. Stegbauer, G. Savasci, F. Podjaski, C. Ochsenfeld and B. V. Lotsch, *Nat. Commun.* **2015**, *6*, 8508.

[30] E. Jin, Z. Lan, Q. Jiang, K. Geng, G. Li, X. Wang and D. Jiang, *Chem* **2019**, *5*, 1632-1647.

[31] Y. Yang, N. Luo, S. Lin, H. Yao and Y. Cai, *ACS Catal.* **2022**, *12*, 10718-10726.

[32] J. L. Sheng, H. Dong, X. B. Meng, H. L. Tang, Y. H. Yao, D. Q. Liu, L. L. Bai, F. M. Zhang, J. Z. Wei and X. J. Sun, *ChemCatChem* **2019**, *11*, 2313-2319.
